# Material Design for Low-Loss Non-Oriented Electrical Steel for Energy Efficient Drives

**DOI:** 10.3390/ma14216588

**Published:** 2021-11-02

**Authors:** Nora Leuning, Markus Jaeger, Benedikt Schauerte, Anett Stöcker, Rudolf Kawalla, Xuefei Wei, Gerhard Hirt, Martin Heller, Sandra Korte-Kerzel, Lucas Böhm, Wolfram Volk, Kay Hameyer

**Affiliations:** 1 Institute of Electrical Machines, RWTH Aachen University, 52062 Aachen, Germany; markus.jaeger@iem.rwth-aachen.de (M.J.); benedikt.schauerte@imf.tu-freiberg.de (B.S.); kay.hameyer@iem.rwth-aachen.de (K.H.); 2 Institute of Metal Forming, TU Bergakademie Freiberg, 09596 Freiberg, Germany; anett.stoecker@imf.tu-freiberg.de (A.S.); rudolf.kawalla@imf.tu-freiberg.de (R.K.); 3 Institute of Metal Forming, RWTH Aachen University, 52056 Aachen, Germany; xuefei.wei@ibf.rwth-aachen.de (X.W.); gerhard.hirt@ibf.rwth-aachen.de (G.H.); 4 Institute of Physical Metallurgy and Materials Physics, RWTH Aachen University, 52074 Aachen, Germany; heller@imm.rwth-aachen.de (M.H.); Korte-Kerzel@imm.rwth-aachen.de (S.K.-K.); 5 Chair of Metal Forming and Casting, TU München, 85748 Garching, Germany; lucas.boehm@utg.de (L.B.); wolfram.volk@utg.de (W.V.)

**Keywords:** electrical steel, loss modeling, tailor-made material design, electrical machines

## Abstract

Due to the nonlinear material behavior and contradicting application requirements, the selection of a specific electrical steel grade for a highly efficient electrical machine during its design stage is challenging. With sufficient knowledge of the correlations between material and magnetic properties and capable material models, a material design for specific requirements can be enabled. In this work, the correlations between magnetization behavior, iron loss and the most relevant material parameters for non-oriented electrical steels, i.e., alloying, sheet thickness and grain size, are studied on laboratory-produced iron-based electrical steels of 2.4 and 3.2 wt % silicon. Different final thicknesses and grain sizes for both alloys are obtained by different production parameters to produce a total of 21 final material states, which are characterized by state-of-the-art material characterization methods. The magnetic properties are measured on a single sheet tester, quantified up to 5 kHz and used to parametrize the semi-physical IEM loss model. From the loss parameters, a tailor-made material, marked by its thickness and grain size is deduced. The influence of different steel grades and the chance of tailor-made material design is discussed in the context of an exemplary e-mobility application by performing finite-element electrical machine simulations and post-processing on four of the twenty-one materials and the tailor-made material. It is shown that thicker materials can lead to fewer iron losses if the alloying and grain size are adapted and that the three studied parameters are in fact levers for material design where resources can be saved by a targeted optimization.

## 1. Introduction

Non-oriented electrical steels are used in the magnetic core of electrical machines. Rolled usually to lamination thicknesses between 0.5 and 0.2 mm, they are electrically isolated, cut and stacked to build up the rotor and stator of the machine. The reduction of the sheet thickness of this iron-based alloy and the addition of silicon and aluminum up to a combined amount of 5 wt % are standard measures to reduce the iron loss of commercial electrical steels. The grades are classified by their thickness and magnetic loss at distinct excitations and specified for example in EN10106 and EN10303. In the European Standard EN10106, “Cold-rolled non-oriented electrical steel strip and sheet”, nominal thicknesses of 0.35, 0.50, 0.65 and 1.00 mm are specified. The grade name M270-50A consists of the information about the maximum allowed iron loss in the first number and information on the sheet thickness in the form of the latter number. The number 50 represents the hundredfold thickness, thus a 0.5 mm steel with a maximum allowed iron loss of 2.7 W/kg at 1.5 T and 50 Hz. For thinner materials, EN10303, “Thin magnetic steel strip and sheet for use at medium frequencies”, can be applied. It specifies the steel grades of 0.05, 0.10, 0.15, 0.20, 0.25, 0.27, 0.30 and 0.35 mm. Thin steel sheets are usually classified in the form of NO20-13, where 20 represents a 0.2 mm sheet and 13 indicates a maximum allowed iron loss of 1.3 W/kg at 1.0 T and 400 Hz. For electric traction drives where higher magnetizing frequencies are expected, sheet thicknesses between 0.2 and 0.35 mm are usually used. On the other hand, certain slow frequency applications, such as large wind generator can have sheets of up to 1 mm.

The application of electrical steel laminations in the soft magnetic core of rotating electrical machines ensures an efficient energy conversion from mechanical to electrical energy or vice versa, if the right electrical steel grade is chosen. As highlighted by the two European standards, a variety of distinct thicknesses for electrical steels are available. The classification lacks in information about the nonlinear material behavior, as the magnetic properties are characterized at the norm points. When machine designers choose one of these grades, it is not ensured that the machine has the same efficiency as an equally classified electrical steel from another supplier.

**Figure 1:**
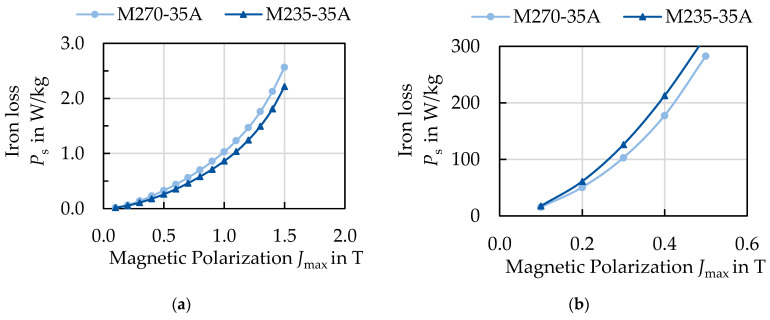
Magnetization curves for two industrial non-oriented electrical steel grades: (**a**) 50 Hz and (**b**) 5 kHz.

**Figure 2:**
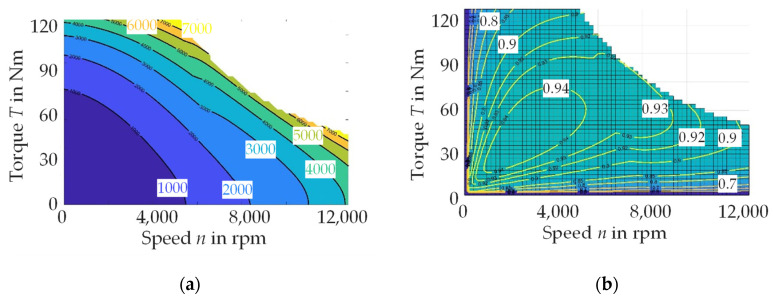
Torque–speed characteristics for a small vehicle: (**a**) loss map in W; (**b**) efficiency map.

The “perfect electrical steel for all applications” is technically not feasible due to the strong nonlinear material behavior of electrical steel and the conflicting requests from the focus of machine design, manufacturing, operation and costs. The approaches to material selection, as presented before, are initially based on the classification and a limited amount of measurement data. Typically, for example, a material classified as M235-35A would be preferred over an M270-50A steel grade. However, this classification represents the iron loss at 50 Hz and the behavior can switch at high frequencies, as shown in Figure 1 for two exemplary electrical steels of these grades. Here, two 0.35 mm electrical steels are displayed at 50 Hz and 5 kHz. At low frequencies, the M235-35A has lower iron losses, as expected by the classification. At 5 kHz, the behavior changes and the M235-35A actually has higher losses. For speed-variable traction drives in mobility applications, the electrical steel is exposed to frequencies between one-digit hertz values and several kilohertz, taking into consideration the magnetizing frequency to reach all operation points in the torque–speed map of the machine, as well as harmonics due to parasitic effects. In Figure 2a the torque–speed map of an exemplary traction drive of a small vehicle is displayed. Speed *n* and frequency *f* of the machine are connected by the pole pair number *p* as follows:

(1) n=fp

**Figure 3:**
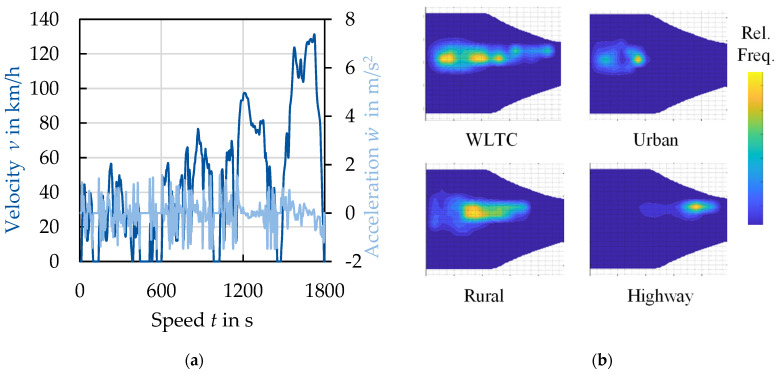
(**a**) Exemplary time–speed map; (**b**) exemplary probability of operation points for different driving cycles in a torque–speed map (WLTC: worldwide harmonized light vehicles test procedure).

In a machine, *p* is fixed by the winding system in the stator. The speed can thereby be varied by a deliberate change of magnetizing frequency. The torque *T* is generated by the relative movement of the rotor to the stator and is proportional to the magnetic flux density in the airgap and thus, directly linked to the magnetization behavior of the electrical steel. At each operation point, the energy conversion causes losses in the excitation windings (copper losses), in the mechanical components (friction losses) and in the electrical steel (iron losses). As a result, each operation point has a different efficiency as displayed in Figure 2b, which is the reason why the overall efficiency of a machine has limited value. The iron loss in the electrical steel is the share of loss that is caused in the electrical steel by the repeated remagnetization process. The presented relations can be summarized as follows: a good magnetization behavior and low loss should be enabled at all relevant operation points for a specific application, but the relevance of operation points is not evenly distributed within the torque–speed map. A driving cycle, as displayed in Figure 3, shows which operation points have a high relevance. A material selection based on these application considerations will lead to the best material choice.

The classification of electrical steel grades based on the magnetic properties is not only insufficient, since the behavior can change at magnetic excitations besides the classification point, e.g., elevated frequency as shown in Figure 1, but it also does not focus on the material itself. The same classification does not mean that it is the same material as it can vary significantly in microstructure, texture or alloying and therefore in magnetic properties. The nonlinear magnetic behavior can be linked to material parameters, such as microstructure, texture, alloying and sheet thickness [1,2]. Usual measures to optimize the magnetic properties are a reduction of the lamination sheet thickness as well as additional alloying with silicon to increase the electric resistivity. Both measures reduce the global eddy current loss. A large grain size reduces coercivity, as well as improving the magnetization behavior, as grain boundaries impede free domain wall motion [2,3]. A rotated-cube texture improves the magnetization and isotropy maximizing the number of easy <100> magnetization axes within the sheet plane [4,5].

Even though these measures improve certain properties, they nevertheless have drawbacks. An increased silicon content reduces saturation polarization due to the decreased share of iron atoms that contribute to magnetization within the alloy. A large grain size increases the excess loss [1,6]. A smaller sheet thickness reduces the filling factor and interferes with processing, e.g., cutting and stacking. Thinner materials are therefore more expensive. A trade-off for each application is necessary to choose the best material. Usually, an excessive focus is placed on the overall efficiency of a machine. Cost and resources can be saved with the right material choice. A detailed consideration between the magnetic properties of the electrical steel and the operating characteristic of electric machines is necessary during the design of electrical machines with some examples being:

- Operating point dependent on excitation conditions: a machine can be designed for a few distinct operating points, e.g., steady industrial applications or the operating conditions can be characterized by a distribution of operating points for speed-variable drives and the entire torque–speed map.
- Different priorities for stationary or mobile applications: a low weight, efficient sizing and a high-power density are especially important if the machine has to be moved, e.g., in electric cars, planes or other mobile applications. The weight and size should be minimized to avoid additional loads.
- Sufficient representation of material parameters in the material models: in order to evaluate the impact of different electrical steel grades during the design of electrical machines, material properties need to be sufficiently represented in the respective models. The effect of microstructure, e.g., can only be evaluated if the effect can be distinguished from the impact of sheet thickness or alloying content.

In this paper, the relations between the relevant material parameters, i.e., sheet thickness, alloying and grain size and non-linear magnetic properties, are studied, with a focus on iron loss. An emphasis is put on the influence that the electrical steel has on the operating behavior of electrical drives and how the performance can be improved by deliberately changing material parameters. The relevance of material design for the application is shown for an exemplary e-mobility case. In order to evaluate the impact of different electrical steels in a simulation study, the iron loss needs to be modeled. The loss model parameters of the semi-physical IEM loss model are identified and correlated with microstructural parameters. A full parametrization with laboratory material enables a chance to create a tailor-made electrical steel.

## 2. Materials and Methods

The studied steel grades in this research are produced on an experimental processing route of hot rolling, cold rolling and annealing as described for the Fe–3.2 wt % Si in [7]. Two iron-based alloys with a nominal content of 2.4 and 3.2 wt % silicon (Si), respectively, were processed to 0.5 and 0.25 mm thin, fully finished electrical steel grades with different grain sizes. A summary of the production process parameters is given in Table 1 and Table 2. A total of 21 final material grades were produced, characterized and studied.

- HT: hot band thickness.
- HS: hot band structure; h = homogenous; b = band structure.
- CR: cold-rolling reduction.
- FT: final thickness after cold rolling.
- AT: annealing temperature.

The metrological characterization of the electrical steel was performed on two 60 mm × 60 mm single sheet testers with two different winding systems for low- and high-frequency measurements. Magnetization curves were obtained with a resolution of 0.1 T between 0.1 and 1.8 T under frequencies between quasi-static excitation up to 5 kHz. The low frequency single sheet tester had 115 primary and secondary windings, whereas the high frequency single sheet tester had 25 windings each. The form factor error was used to evaluate the quality of the measurements to ensure comparability. For sinusoidal excitation, the form factor was 1.11. An error below 1% ensured high comparability.

The grain size was obtained by the line intercept method in different layers of the rolling direction (RD)–transverse direction (TD) plane. One layer was on the surface and one in the middle of the sheet thickness. The final surface finish was achieved with a 1 µm alcohol-based diamond suspension. Surface sections were etched with 5% Nital and evaluated on a light optical microscope. More than 300 grains were measured for each layer and direction. In order to account for the anisotropy, grains were measured in the RD and TD. The layers were weighted to resemble the grain size distribution across the thickness of the sheet with both the sheet center and surface being displayed in Table 3. As the middle plane was considered to be representative for the bulk material, the middle was weighted twice compared with the surface microstructure. The weighted results of the measurements in the RD, TD and averaged orientation are displayed in Table 4.

For texture measurements, an X-ray diffraction was performed. The sample size for the measurements was 120 mm^2^, with a measurement area of 64 mm^2^ which corresponds to several thousand grains for typical NO electrical steel microstructures. Texture measurements were done in two layers, identical to the grain size measurements, with the same weighting proportions. In order to quantify the texture, the so-called *A*-parameter [5] was used. It relates the crystallographic texture by evaluating the distribution of easy magnetization axes with respect to the sample geometry. The *A*-parameter describes the mean angle of all orientations of the orientation distribution function between the magnetization vector and the closest easy axis of an orientation. In Table 4, the *A*-parameters in the RD, TD and averaged orientation are displayed. The averaged parameter is a mean value of all orientations between the RD and TD in 5° steps to account for hard magnetization axes in between.

## 3. Magnetic Properties of the Processed Materials

**Figure 4:**
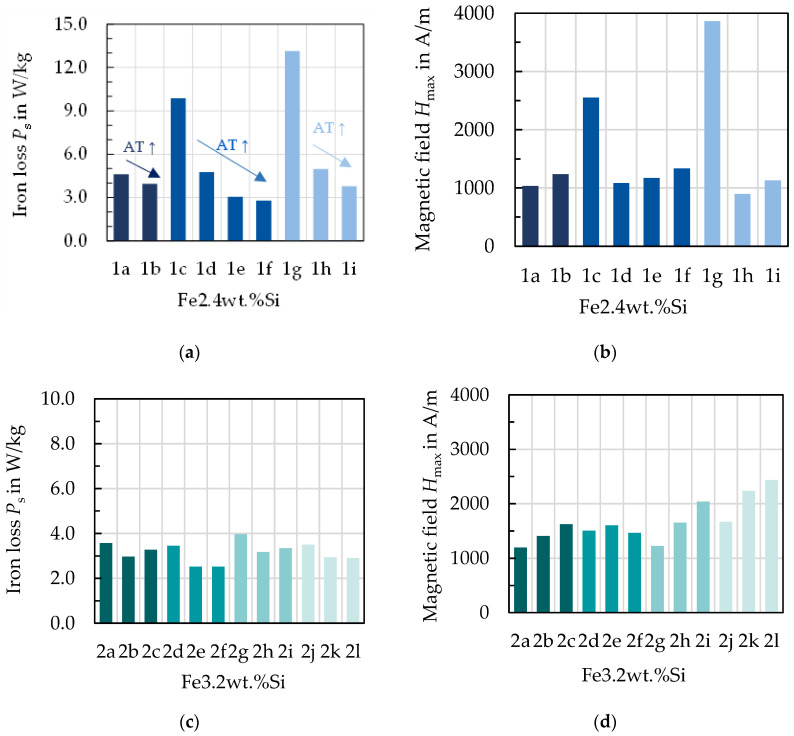
Magnetic properties of the studied material grades at 1.5 T and 50 Hz, with the same color indicating that only annealing temperature (AT) as highlighted in (**a**) has changed for these states: (**a**) loss for Fe–2.4 wt % Si; (**b**) magnetization for Fe–2.4 wt % Si; (**c**) loss for the Fe–3.2 wt % Si; (**d**) magnetization for Fe–3.2 wt % Si.

**Figure 5:**
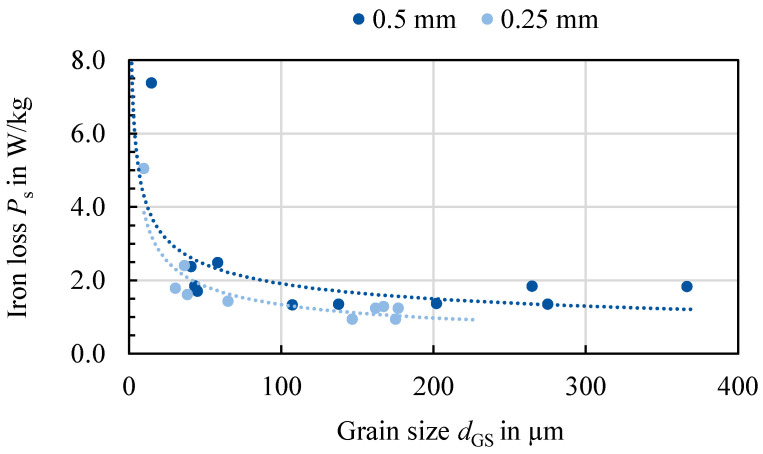
Iron loss at 1.0 T and 50 Hz as a function of grain size for all studied materials sorted by sheet thickness.

**Figure 6:**
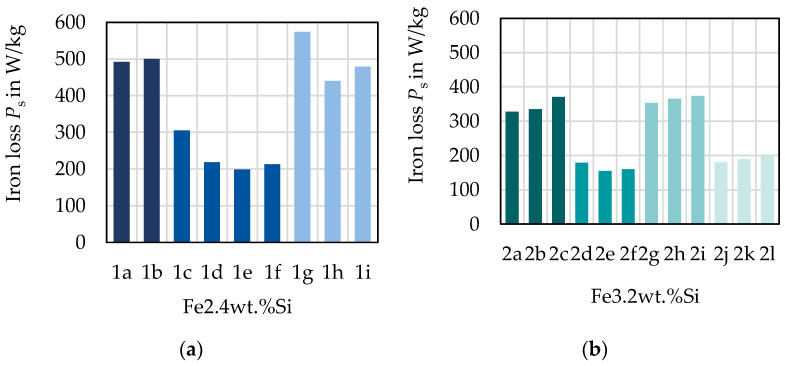
Iron loss at 1000 Hz for all studied 21 materials at 1.5 T: (**a**) Fe 2.4 wt % Si; (**b**) Fe 3.2 wt % sSi.

The magnetic properties vary for the produced steel grades due to the processing parameters and their effect on the microstructure and texture as well as on the sheet thickness. In Figure 4, Figure 5 and Figure 6 exemplary results are presented for selected excitation points. A few aspects of the material parameters must be considered during the interpretation of the results. Generally, the processing parameters during the production of the Fe–2.4 wt % Si lead to a larger range of grain sizes after annealing compared with the grain sizes obtained for the Fe–3.2 wt % Si. Two samples, 1c and 1g, were not fully recrystallized after annealing. Furthermore, the grain size for the 0.25 mm sheets was generally smaller after annealing at higher temperature compared to the 0.5 mm. This is likely caused by surface effects, when the grain size is approaching the dimension of approximately half the sheet thickness. Due to the large number of results, the following diagrams only show magnetic properties in the RD. Consequently, when correlating with grain size, the grain size in the RD is used.

In Figure 4, the maximum magnetic field strength $H_{\max}$ and the iron loss $P_{S}$ at 1.5 T and 50 Hz are displayed for all samples. In Figure 4a,c, the iron loss is presented for both alloys. The same-colored bars represent the material states of the same alloy, hot band state, hot rolling and cold rolling conditions, where only the annealing temperature (AT) has increased. For all but two states, an increased annealing temperature leads to a lower loss at 50 Hz and 1.5 T. The not fully recrystallized states show significantly higher loss. For 2c and 2i, which both are 0.5 mm thick, have the same silicon content and are annealed at 1100 °C, the loss increases compared to their counterpart annealed at 1000 °C. According to [8], the loss at low frequencies is dominantly affected by grain size. This can be confirmed for the studied materials as displayed in Figure 5. Noticeably, the iron loss decreases with increasing grain size. The samples are divided into the 0.25 mm and 0.5 mm samples, as part of the losses is attributed to the so-called Foucault eddy current loss which are proportional to the sheet thickness. Moreover, it is evident that for the 0.25 mm samples, the grain size is smaller. The strongest decrease of iron loss occurs at grain sizes up to 50 and 100 µm. From there on, the grain size affects the iron loss to a small extent. These results are congruent with the literature [8]. The hysteresis component, which is dominant at low to medium frequencies as presented in [6], has the same course. In addition, the results in [9] show the same course for various low to medium frequencies, with a steep increase of loss for small grain sizes below 100 µm, but nearly unaffected loss dependence above this grain size. The static hysteresis component is affected by grain size, as grain boundaries impede domain wall movement leading to a larger coercive field strength, and thus a wider hysteresis curve [3]. The area of the static hysteresis curve is directly related to the hysteresis loss component. At higher frequencies, the Foucault eddy current loss and additional excess losses start to affect the total loss decisively. The relation that an increase of annealing temperature decreases the loss is not valid anymore, as presented in Figure 6 at 1 kHz. Due to a higher share of the other loss components, this trend has changed. The additional loss component is related to the microstructure, but in a contrary relation compared with the hysteresis loss. The Foucault eddy current loss is mainly related to the thickness and alloying. The direct proportionality of the Foucault eddy current loss to the sheet thickness can be seen clearly in Figure 6a,b as the 0.25 mm sheets (1c–f, 2d–f and 2j–l) show distinctly smaller overall loss.

The magnetization behavior for the studied materials is displayed in Figure 4b,d. For the not fully recrystallized samples, the magnetic field strength to reach 1.5 T at 50 Hz is very large. Generally, the Fe–2.4 wt % Si alloy requires less magnetic field strength for an induction of 1.5 T at 50 Hz compared with the Fe–3.2 wt % Si alloy, which is the result of less iron in the alloy. For all samples, except the ones not fully recrystallized, and 2d–f, the magnetic field increases with annealing temperature and thus with grain size. This was not previously expected, as larger grains are generally regarded as beneficial for the magnetization process due to less domain wall pinning [2,3]. However, similar results were obtained by [10,11], where magnetization was also diminished with larger grains on experimental material.

**Figure 7:**
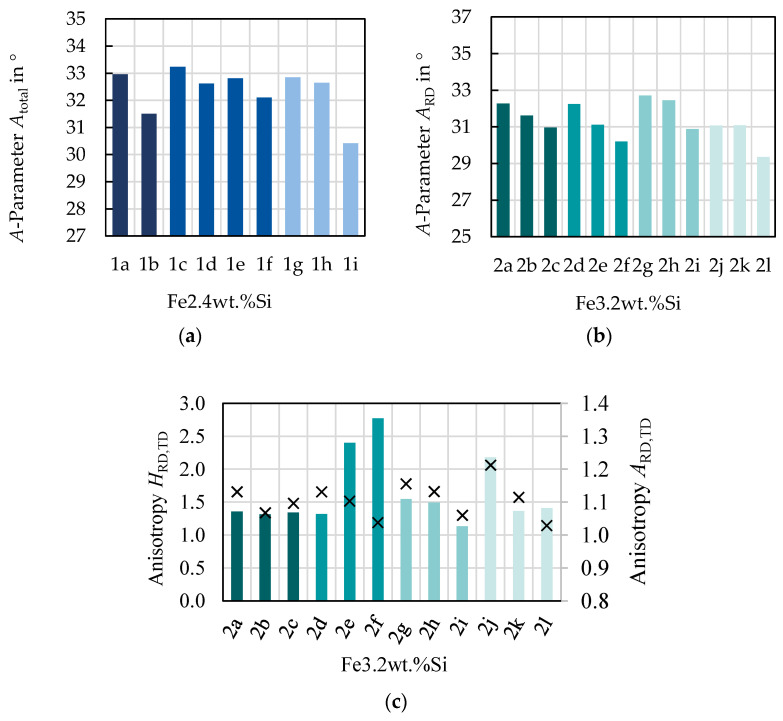
Evaluation of the *A*-parameter and magnetization behavior: (**a**) mean *A*-parameter for Fe–2.4 wt % Si; (**b**) mean *A*-parameter for Fe–3.2 wt % Si; (**c**) magnetic field at 1.0 T, 50 Hz with bars representing maximum magnetic field strength and × symbols representing *A*-parameters.

Alloying and grain size are not the only parameters with an influence on the magnetization. The texture also has to be considered as a possible explanation for the magnetization behavior. However, as presented in Figure 7a,b, the texture improves with higher annealing temperature. The *A*-parameter decreases with higher annealing temperature; thus, the grains are more favorably oriented, with easy magnetization axes closer to the magnetization direction. As a result, the magnetization should theoretically be easier as the alloy and sheet thickness are the same and grain size and favorable texture increase with higher annealing temperature. However, this is not the case. A third explanation could be residual stress, induced during the processing of the material that remains in the samples as the magnetizability is very sensitive to complex mechanical stress states [12]. Due to the laboratory processing route, the rolling as well as the annealing conditions cannot be directly compared to industrial processing. For example, there is no strip tension during rolling and the annealing is a discontinuous process. This could lead to mechanical residual stresses and affect the properties. The higher the final annealing temperature, the more important the cooling conditions become. A slow cooling might reduce the residual stress; however, it would also affect grain growth.

Although, crystallographic texture has no dominant effect on the magnetization behavior for the studied samples, it affects the magnetic anisotropy. In [6], this was shown for the Fe–2.4 wt % Si alloy. In Figure 7c, the trend between magnetization anisotropy and crystallographic anisotropy is displayed for the Fe–3.2 wt % Si alloy. With the exception of 2d–2f, the relative change in *A*-parameter from RD to TD, marked as × symbols, follows the same trend as the relative magnetization anisotropy $H_{\mathrm{RD},\mathrm{TD}}$. All in all, most of the magnetization behavior can be linked to material properties and these relations can be used for the modeling of the magnetic behavior.

## 4. Loss Modeling, Parameter Identification and Tailor-Made Approach

There are various approaches to model the iron loss of electrical steels [13,14]. They range from solely mathematical descriptions to descriptions of the entire magnetization process in hysteresis models or to physically motivated analytical descriptions. In order to evaluate the effect of different materials on the operating characteristics of electrical machines, a loss model should be easy to implement in finite element simulations and at the same time, be detailed enough to allow conclusions on the differences between the studied materials. The semi-physical IEM loss modeling approach allows a physical interpretation of the simulated results [15]. It is based on the loss separation of the Bertotti model, which separates the total loss into a static, a classical Foucault eddy current and an excess component [16]. However, as shown in [15,17], the classical Bertotti model underestimates iron losses at high magnetic flux densities and high frequencies that are linked to the neglect of saturation induction [15]. As many electrical machines operate to a large extent in this region, an adaptation of the Bertotti approach has been formulated as the IEM-Formula. The model describes the total loss $P_{\mathrm{iron},\mathrm{IEM}}$ as a function of the magnetic flux density *B*_max_ and magnetizing frequency *f*. The IEM-Formula shows an improvement in model accuracy at high magnetic flux densities and high frequencies [14]. Due to a fourth loss term with a higher order dependence added to the loss components of the Bertotti model, the following mathematical formulation results:

(2) $$P_{\mathrm{iron},\mathrm{IEM}}=\begin{array}{c} \underset{︸}{a_{1}B_{\max}^{\alpha +B_{\max}\beta }f}+\underset{︸}{a_{2}B_{\max}^{2}f^{2}}+\underset{︸}{a_{2}a_{3}B_{\max}^{a_{4}+2}}+\underset{︸}{a_{5}{\left(B_{\max}f\right)}^{1.5}}\\ P_{\mathrm{hyst}}\;\;\;\;\;+\;\;\;\;\;\;\;P_{\mathrm{cl}}\;\;\;\;\;+\;\;\;\;\;\;P_{\mathrm{nl}}\;\;\;\;\;\;+\;\;\;\;\;\;P_{\mathrm{exc}} \end{array}$$

The added term is called saturation or nonlinear loss $P_{\mathrm{nl}}$. In this equation, $a_{I}$ as well as α and β are the identified material parameters. The parameter identification process is based on the statistical loss theory as described in [18]. In order to identify the parameters $a_{1}$, α and β, the energy $E_{\mathrm{DC}}$ consumed during quasistatic measurements is measured as:

(3) $$E_{\mathrm{DC}}=a_{1}B_{\max}{}^{\alpha +B_{\max}\beta }$$

The classical Foucault eddy current loss component parameter $a_{2}$ can be approximated by the following Equation (4) with information on the sheet thickness *d*, specific density *ρ* and specific electrical resistivity *ρ*_el_,

(4) $$a_{2}=\frac{\pi^{2}d^{2}}{6\rho \rho_{\mathrm{el}}}$$

The excess loss parameter $a_{5}$ can be identified by measurements at relatively low magnetic flux densities and frequencies or calculated from material-dependent properties [16,18]. The identification of $a_{5}$ with material parameters is based on the theory of magnetic objects [16]. The physical interpretation relates to local eddy currents induced in the vicinity of, e.g., grain boundaries due to the movement of domain walls and subsequent change of d*B*/d*t*. For the identification without material parameters, the excess loss term is separated from low-frequency measurements (3–10 Hz) by subtracting the hysteresis (Equation (3)) and the classical Foucault eddy current losses (Equation (4)). The saturation losses are neglected in this case, as they are identified last. The parameters $a_{3}$ and $a_{4}$ are parametrized mathematically from the measured loss that remains after the identification and subtraction of the hysteresis, classical Foucault eddy current and excess loss components.

**Figure 8:**
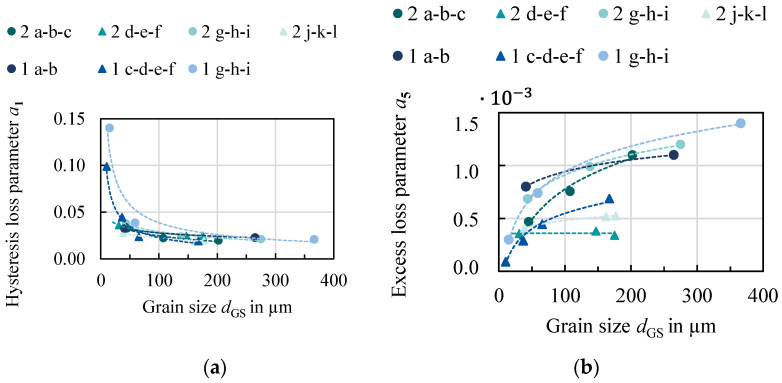
Loss parameters identified for the IEM model for all studied materials: (**a**) hysteresis loss parameter; (**b**) excess-loss parameter. Triangles and squares represent 0.25 mm and 0.5 mm, respectively.

The loss parameters for all studied samples were identified as well as evaluated and are discussed in the following section. The electric resistivity can be calculated or taken from diagrams according to [19] or [20] based on the silicon and aluminum content. As the relation between the Foucault loss, the silicon and aluminum content and the sheet thickness are solely calculated, the correlations of $a_{2}$ are not displayed. In Figure 8, the hysteresis and excess loss parameters are displayed as a function of grain size. The circular markers represent the 0.5 mm grades and the triangular markers indicate the 0.25 mm grades.

The hysteresis loss decreases with increasing grain size as previously observed and discussed in the literature and in Section 3. The excess loss, on the other hand, shows an opposite trend. With increasing grain size, excess loss increases. This can be explained by the physical interpretation that grain boundaries impede domain wall movement which leads to less induced local eddy currents. The effect of grain size on the hysteresis as well as on the excess loss is especially strong in the range up to 150 µm. After that, the slope decreases. What can also be observed is the impact of sheet thickness. For both the hysteresis and the excess loss, smaller sheet thickness is beneficial. The effect is more pronounced for the excess loss. The nonlinear loss component shows no distinct correlation to either grain size, sheet thickness or alloying.

In order to utilize such results in electrical machines applications, the feasibility of a tailor-made material design approach was considered. The aim was not to choose a material from the 21 produced laboratory grades, but to use the relations between the loss parameters and material parameters to design an improved steel grade for an e-mobility application of a small vehicle (Section 5). The application requirements were generalized in this case and not tailored to a specific drive cycle. This simplification was made to allow a comparison between the different produced steel grades and to highlight the influence of these materials on the different areas of the torque–speed map. Therefore, neither high- or low-speed operation points were weighted individually. The tailor-made approach was applied to study whether a deliberate design of loss parameters leads to a significant change of loss in the expected ranges of the torque–speed map.

**Figure 9:**
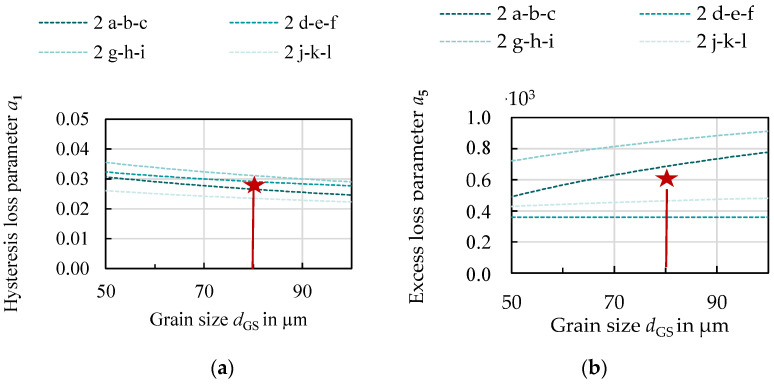
Detail from Figure 8 to determine optimum grain size for a 0.3 mm, Fe–3.2 wt % Si: (**a**) hysteresis loss parameter; (**b**) excess-loss parameter.

With the above gained knowledge (Equation (4), Figure 8), as well as continued considerations, a material was designed. Due to the beneficial effect on the iron loss, the 3.2 wt % Fe–Si alloy was chosen. Both alloying concepts have to be treated as separate parameter spaces that can be interpolated. If more experiments had been performed with more alloys, the effect of alloying could have been considered. With only two alloys this was not possible. A sheet thickness of 0.3 mm was chosen due to the trade-off between magnetizability, production, processing and iron loss. From Figure 9, a grain size that ensures a combined minimum of excess and hysteresis loss was chosen. In Table 5 the resulting loss and material parameters for the newly designed material are displayed. It must be noted that no optimization algorithm was used for this, but a feasibility approach that considered the loss parameters, processing effort and costs. The purpose of this example is to highlight how a tailor-made material design can be enabled and how it affects the electrical machine, as presented in the following Section 5. The parameters *α*, *β*, $a_{3}$ and $a_{4}$ were chosen from diagrams analogous to Figure 9 for all samples of the Fe–3.2 wt % Si alloy, but they did not show a distinct trend. Parameters that are not linked to the loss model, but to a machine model, i.e., the filling factor, have to be considered as well. Smaller sheet thickness leads to a higher proportion of the isolation coating in the axial length of the machine. It also makes the assembling of the machine more difficult.

## 5. Simulation Study Results and Discussion

In order to highlight the effect of different materials on the operational characteristic of electrical machines, a simulation study was performed. Due to various produced materials and the subsequent large number of results, the simulation was only performed on the four materials 1b, 2g, 1d and 2k out of the 21 grades that represent the various design strategies for electrical steel: the alloying with silicon, a thickness reduction and microstructure optimization.

**Figure 10:**
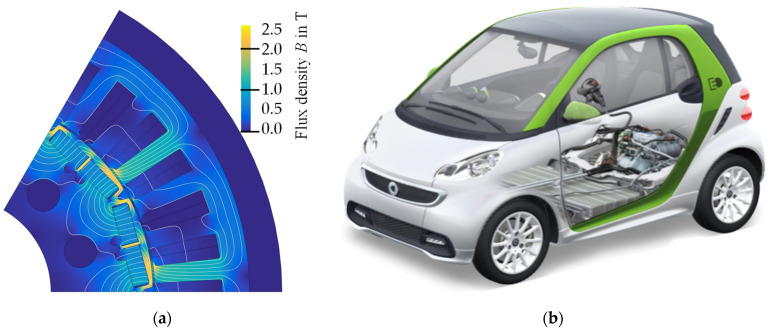
(**a**) One exemplary flux solution of the studied PMSM; (**b**) vehicle in which the studied PMSM is used.

As an example, a permanent magnet synchronous machine (PMSM) of a small electric vehicle (Figure 10b) was studied. A PMSM is a typical traction drive of modern electric vehicles, and this one is designed for a standard 400 V board grid. The PMSM features a rated power of 55 kW at approximately 7200 rpm, while a peak power of over 80 kW and a peak torque of 125 Nm can be reached within the current limit of 300 A. This motor was chosen due to the high electrical base frequency of 1200 Hz, which is on par with modern traction drives of a higher rated power or higher maximum speed and allows for a meaningful comparison of soft magnetic materials.

The simulation study was carried out with a state-of-the-art approach. One pole pair of the machines’ cross section was modeled and the magnetic flux calculated via 2D finite element analysis (FEA). The parameter space spanned the rotational steps as well as the current in the direct and in the quadrature axis. On the one hand, magnetic hysteresis was neglected due to its integration in the FEA being a topic of ongoing research, and on the other hand, eddy currents were a valid aspect in assuming buried magnet machines; thus, the respective time steps were assumed to be independent from each other. The magnetization curve used in the FEA was specific to the material, so the FEA had to be performed for each considered material. Based on the FEA results (example shown in Figure 10 on the left), a torque map in the d-q-space was generated, which in turn was used to calculate the optimal current vectors for each speed using the max-torque-per-ampere (MTPA) method.

The magnetization behavior of the studied materials was represented in the simulation by the reluctivity, i.e., the magnetic field strength *H*, as a function of the magnetic flux density *B*. The reluctivity ν was used to solve the vector-potential during the finite-element magnetic field simulation. As the magnetic properties of the materials were calculated, values for the reluctivity far in the saturated region were needed. Therefore, an extrapolation of the magnetic properties beyond the measurement range was applied. This extrapolation to the saturation was based on the saturation magnetic flux density and calculated according to [21]. The permeability and saturation magnetization were thereby considered in the simulation.

The iron losses in the rotor and stator core were calculated element-wise based on the flux solution using the rotational IEM-Formula [22] with the loss parameters which were identified for the specific material.

For the evaluation of an efficiency map as depicted exemplarily in Figure 2, the total losses, which are the sum of iron, friction and ohmic losses, must be known. To calculate the friction and ohmic losses in absence of more advanced thermal, mechanical and fluid flow models, some justified assumptions had to be made. The easiest and with the least impact were assumptions about the friction losses, which were assumed to be proportional to the square of the speed and estimated to reach 100 W at the maximum speed of 12,000 rpm. To scale the ohmic resistance of the winding to calculate the copper losses, the winding temperature is necessary, which depends on the cooling system. It was assumed that the steady state winding temperature is proportional to the winding losses, and partly to the losses in the stator iron that surrounds the slots and in the rotor iron, whose losses are dissipated mainly through the stator cooling (compare Equation (5)).

(5) $$T_{\mathrm{winding}}\;\sim \;\left(P_{\mathrm{loss},\mathrm{copper}}+k_{\mathrm{temp},\mathrm{ironlosses}}\cdot P_{\mathrm{loss},\mathrm{iron}}\right)\;$$

To conclude from the losses to the winding temperature, two fix points were necessary. The first one was trivial as ambient temperature of 20 °C sits at zero losses. For the second one, the steady state winding temperature at the rated torque of 80 Nm and zero speed was estimated to be 100 °C. The impact-factor of the iron losses was set to 70%. Both values were tuned to achieve realistic torque–speed maps comparable to the measurements of the real machine. Equation (5) is a simple differential equation, which is solved numerically and quickly converges within few iterations.

**Figure 11:**
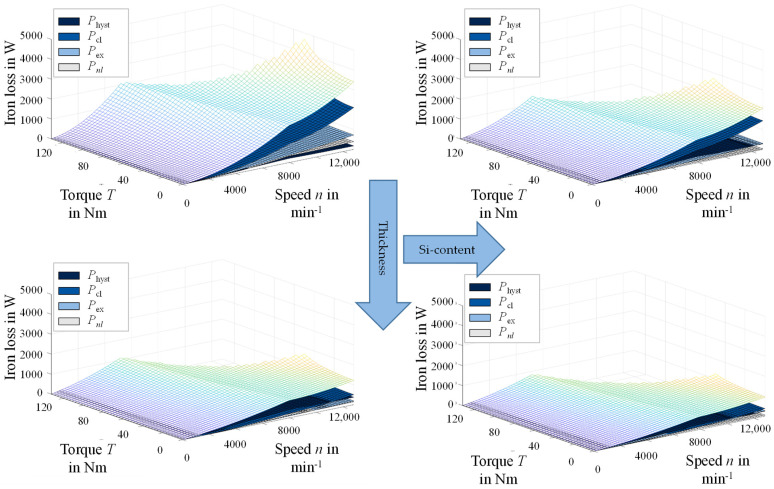
Simulation study results of total iron loss in a torque–speed map: (**a**) 1b— Fe–2.4 wt % Si, 0.5 mm, 265 µm; (**b**) 2g–Fe 3.2 wt % Si, 0.5 mm, 43 µm; (**c**) 1d—Fe–2.4 wt % Si, 0.25 mm, 37 µm; (**d**) 2k—Fe–3.2 wt % Si, 0.25 mm, 162 µm, nomenclature according to Table 1, Table 2 and Table 3.

In Figure 11, the iron loss is shown as a function of the torque–speed characteristic for the four materials. Figure 11a,b shows the results for 0.5 mm steel of both alloys. Figure 11c,d shows results for the 0.25 mm steel. On the left side of Figure 11, the materials with the Fe–2.4 wt % Si are displayed (Figure 11a,c), whereas the Fe–3.2 wt % Si are displayed on the right side (Figure 11b,d). The results show that the different processing and resulting materials have a significant effect on the total iron loss over the entire torque–speed map. The slope in the low-speed high-torque area, the field weakening region and maximum torque or speed are different for each material. A general trend towards a decrease of iron loss with decreasing thickness and increasing silicon content, as highlighted in Figure 11, can be observed. A detailed look at the loss component distribution allows further insight to the previously discussed correlations with material parameters.

**Figure 12:**
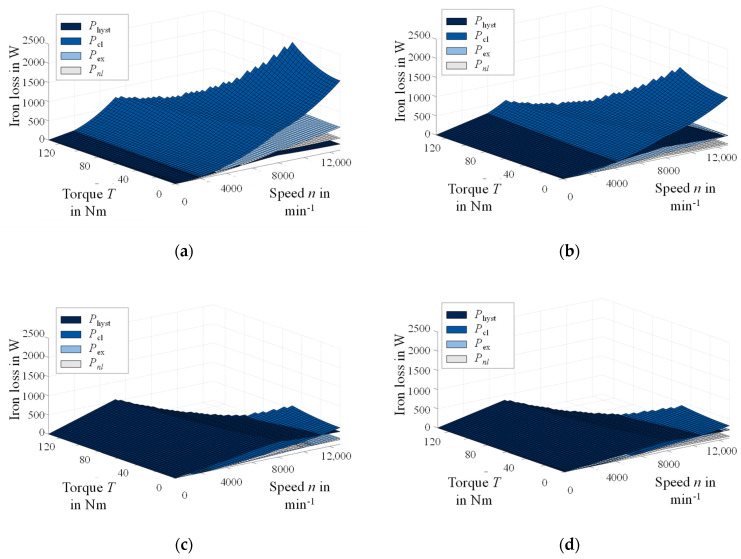
Simulation study results of stator loss in a torque-speed map: (**a**) 1b—Fe–2.4 wt % Si, 0.5 mm, 265 µm; (**b**) 2g—Fe–3.2 wt % Si, 0.5 mm, 43 µm; (**c**) 1d—Fe–2.4 wt % Si, 0.25 mm, 37 µm; (**d**) 2k—Fe–3.2 wt % Si, 0.25 mm, 162 µm.

In Figure 12, the stator loss for the four materials is displayed separately into the four loss components. The classical Foucault eddy current loss decreases with increasing silicon content and decreasing thickness, which is observable/noticeable in a direct comparison of Figure 12a–c. The hysteresis loss is smallest for the largest grain size (Figure 12a) and generally decreases with increasing grain size, i.e., Figure 12c has higher hysteresis losses than Figure 12b,d. It becomes evident as well that with decreasing thickness, hysteresis loss becomes dominant in a far greater area of the loss map compared with the 0.5 mm materials, where classical Foucault eddy current loss is dominant. For thin materials, a grain size optimization can potentially become more important, but simultaneously, large grains are harder to obtain due to the limitation imposed by the thickness, as discussed in Section 3. In Figure 12a,b, the reverse effect of increasing grain size on the excess loss can also be observed with Figure 12b, having distinctly lower excess loss compared to Figure 12a, which is in accordance with the results of Section 4 and the scientific literature.

**Figure 13:**
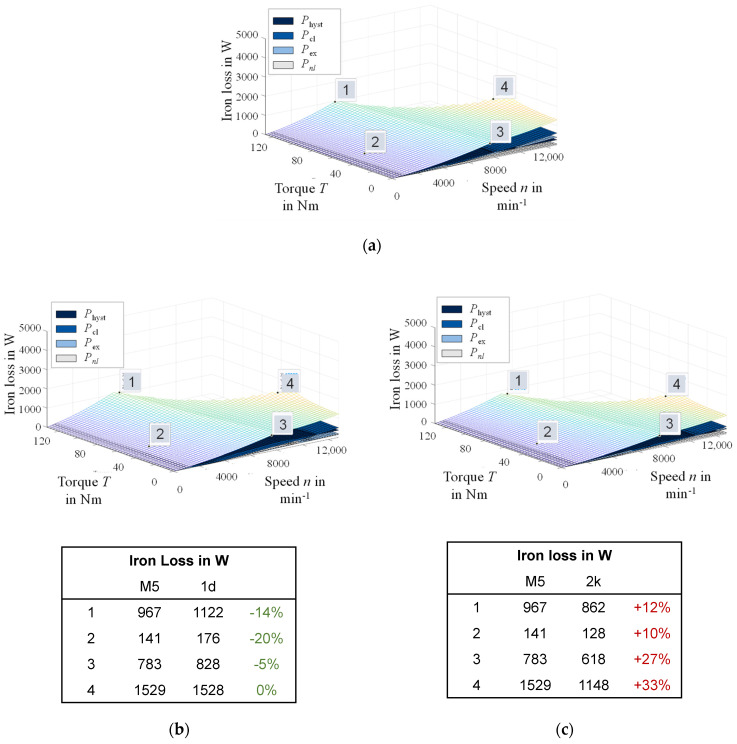
Simulation study results and comparison of the tailor-made material (M5) with two 0.25 mm steel grades in a torque–speed map: (**a**) Tailor-made material, Fe–3.2 wt % Si, 0.3 mm, 80 µm; (**b**) 1d—Fe–2.4 wt % Si, 0.25 mm, 37 µm; (**c**) 2k—Fe–3.2 wt % Si, 0.25 mm, 162 µm.

The simulation study was performed on the tailor-made material (M5) designed in Section 4 and is now compared to the performance of the experimental grades. In Figure 13, the results are displayed and directly compared to the two 0.25 mm sheets that showed distinctly better magnetic properties compared with both 0.5 mm steel grades and are therefore discussed. The tailor-made material has a thickness of 0.3 mm, an alloying content of Fe–3.2 wt % Si and a grain size of 80 µm. The direct comparison is done on four exemplary operating points in different ranges of the application. Compared with the material 1d, the tailor-made material has larger grains, a higher alloying content, but also an increased thickness. The loss of the tailor-made material is better at all operating points. The design approach shows that this material is beneficial for the studied application.

Compared to the material 2k, the tailor-made material, however, has a higher loss at all operating points. The reason is mainly due to the increased classical Foucault eddy current loss. As a result, the loss increase is especially pronounced at high speeds where the share of this loss component is highest, i.e., at operating points three and four. The tailor-made material cannot directly compete with material 2k in terms of magnetic properties. However, a few aspects have to be considered. First, the results show that a material with a higher sheet thickness can compete with thinner materials in general, as seen in the comparison between 1d and the tailor-made material. A further optimization of alloying to a higher Si content could counter the effect of the Foucault eddy current loss and improve the properties of the tailor-made material in direct comparison with 2k. Moreover, since the thickness ratio of sheet thickness to isolation coating has not been considered separately in the magnetic field simulation, the tailor-made material has a further systematic disadvantage in direct comparison. Greater sheet thickness is not only less expensive but also easier to process and produce, which becomes important if the alloying is increased. A Fe–3.2 wt % Si is harder to cold roll due to the increased strength and brittleness. From a manufacturing point of view, a reduction of thickness with less alloying or an increase of alloying with a higher sheet thickness could both be considered to optimize the material. With a smaller sheet thickness, the grain size optimization is also more important, due to the increased share of the hysteresis component of the total loss. A closer look at the potential of tailor-made material design from the viewpoint of magnetic properties, processing and economic considerations can be deduced from the presented results. The effect a material design can have on the application is evident and further studies can improve the tailor-made approach.

## 6. Conclusions

In this paper, various aspects of the material design of non-oriented electrical steels have been discussed. By means of 21 experimentally produced steel grades of two alloys processed with hot rolling, cold rolling and final annealing, the interrelations between the relevant material parameters, i.e., sheet thickness, grain size and alloying, as well as the magnetic properties, have been characterized. The influence of different materials on the operating characteristic of an electrical machine has been highlighted with a simulation study with four out of the twenty-one produced grades and one tailor-made material for an electric vehicle traction drive. The general conclusions of this study can be summarized as follows:

- The processing of the materials and resulting variation of material properties have a significant effect on the application. Sheet thickness, grain size and alloying are the dominant parameters that can be deliberately changed during the production process. These parameters influence the loss component distribution in the torque–speed map. Thus, material design for low-loss non-oriented electrical steel for energy-efficient electrical drives need to consider the application requirements to account for the operating-point-dependent iron losses and required efficiency.
- A saving of material, resources, production costs or energy can be enabled, with a consideration of interdependencies. Two examples are: grain size optimization is more important for thinner sheets, as hysteresis loss has a greater share on the total iron loss; a higher thickness reduction is easier with lower alloying content whereas a higher thickness with more silicon and aluminum could lead to lower overall loss depending on the machine. Thereby, fewer steel sheets are required for the machine and manufacturing becomes easier.

The results were chosen exemplarily to highlight the relations and considerations when selecting materials for specific applications. Further studies are required to define a structured design routine based on the results. One aspect that should be studied is the consideration of driving cycles and the formulation of required operating points from the perspective of the machines. The magnetic induction and frequency distribution should be quantified and evaluated so that loss modeling with different parameters and an optimization algorithm can be used. The driving cycles become especially crucial for the optimum grain size determination. With an increasing number of operating points in the high-speed area, the excess loss becomes more important compared with the hysteresis loss. As a result, in the case of low-frequency applications, i.e., in urban drive cycles, an emphasis on the hysteresis loss is beneficial. Furthermore, additional studies on the impact of silicon content and other alloying elements should be studied to improve the material design parameters.

## Figures and Tables

**Table 1:** Overview and sample names of the processing routes for the Fe–2.4 wt % Si.

Sample Name	1a	1b	1c	1d	1e	1f	1g	1h	1i
Si in wt %	2.4	2.4	2.4	2.4	2.4	2.4	2.4	2.4	2.4
HT in mm	1.0	1.0	1.0	1.0	1.0	1.0	2.4	2.4	2.4
HS	h	h	h	h	h	h	h	h	h
CR in %	50	50	75	75	75	75	80	80	80
FT in mm	0.5	0.5	0.25	0.25	0.25	0.25	0.5	0.5	0.5
AT in °C	1000	1200	800	900	1000	1200	800	1000	1200

**Table 2:** Overview and sample names of the processing routes for the Fe–3.2 wt % Si.

Sample Name	2a	2b	2c	2d	2e	2f	2g	2h	2i	2j	2k	2l
Si in wt %	3.2	3.2	3.2	3.2	3.2	3.2	3.2	3.2	3.2	3.2	3.2	3.2
HT in mm	1.0	1.0	1.0	1.0	1.0	1.0	1.0	1.0	1.0	1.0	1.0	1.0
HS	h	h	h	h	h	h	b	b	b	b	b	b
CR in %	50	50	50	75	75	75	50	50	50	75	75	75
FT in mm	0.5	0.5	0.5	0.25	0.25	0.25	0.5	0.5	0.5	0.25	0.25	0.25
AT in °C	900	1000	1100	900	1000	1100	900	1000	1100	900	1000	1100

**Table 3:** Micrographs of the RD–TD plane for all studied materials.

1a		1b		1c	
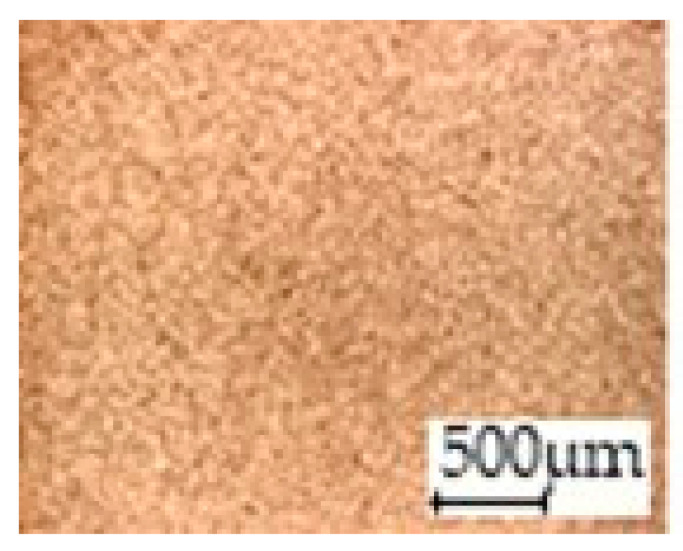	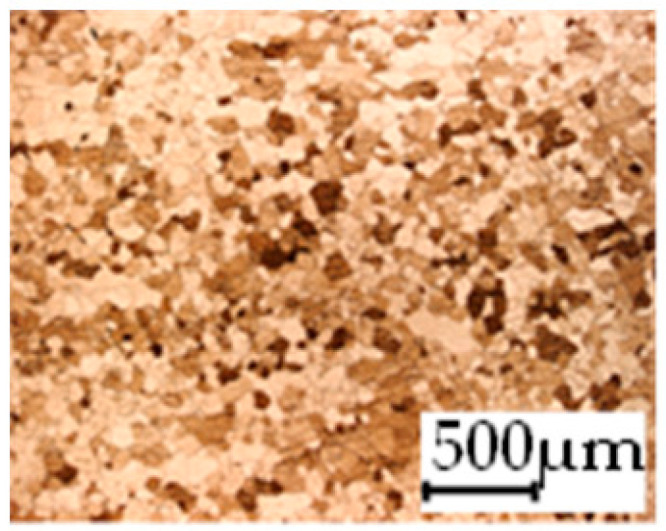	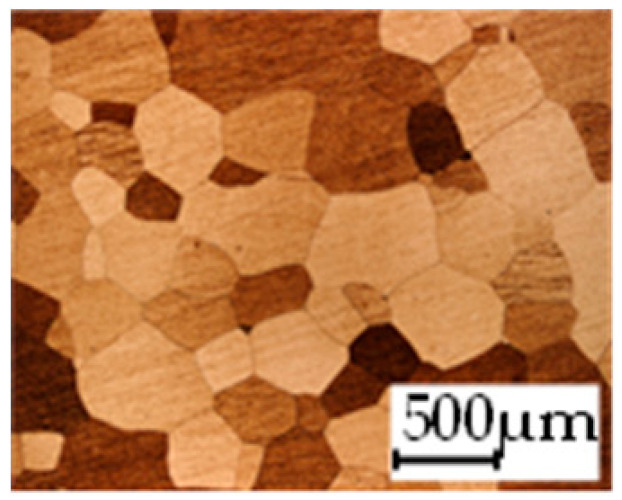	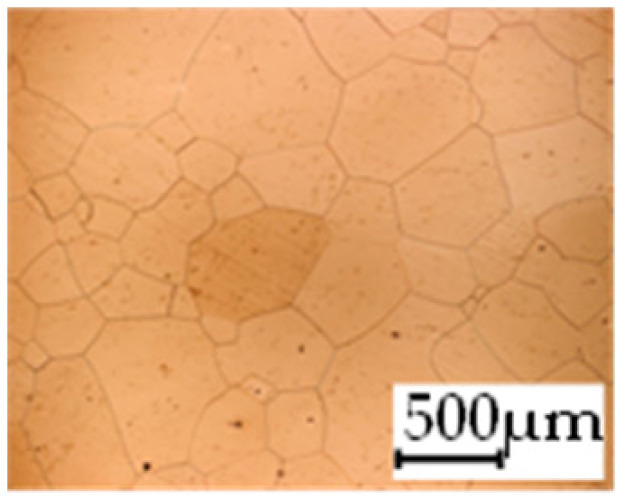	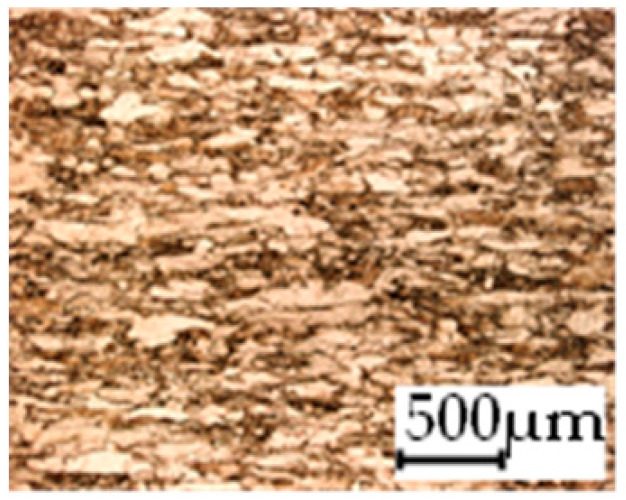	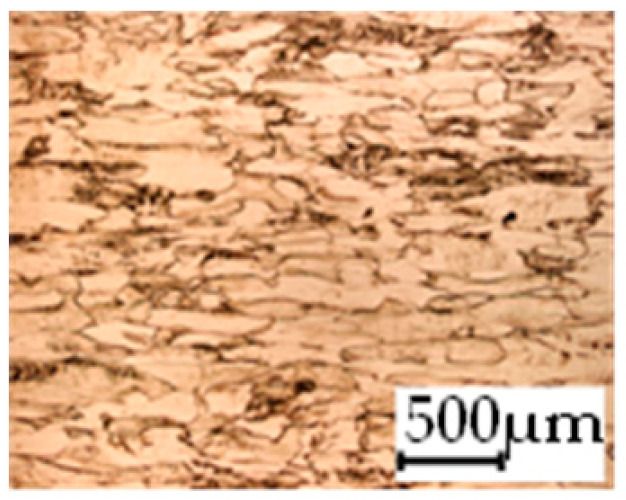
Surface	Middle	Surface	Middle	Surface	Middle
1d		1e		1f	
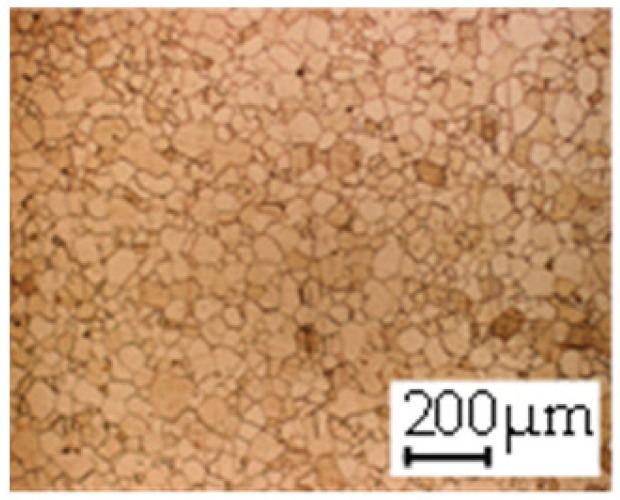	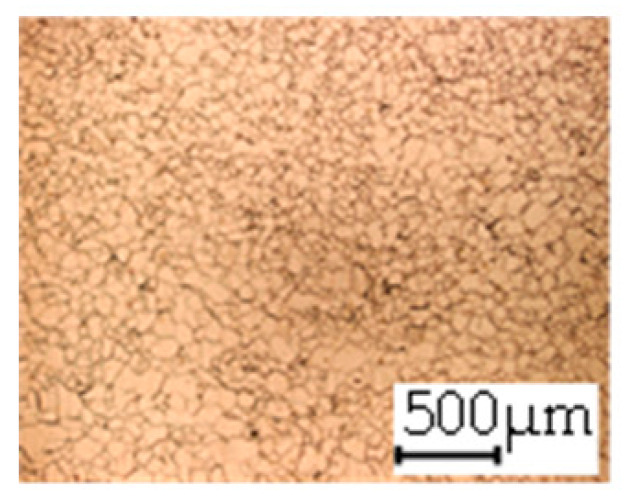	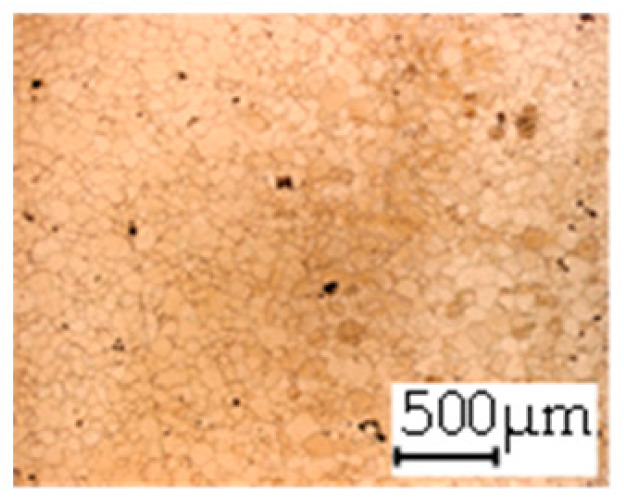	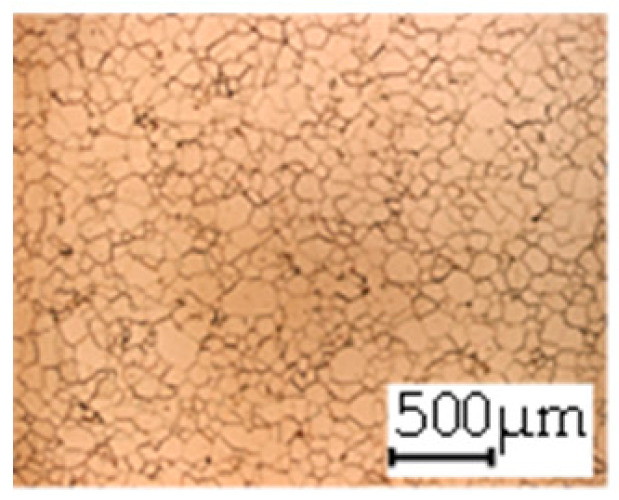	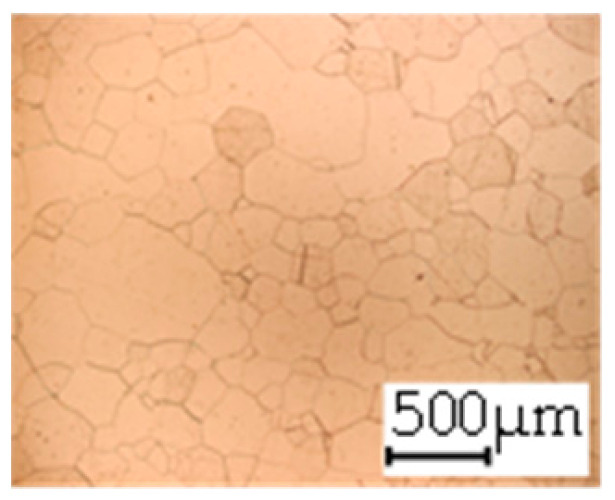	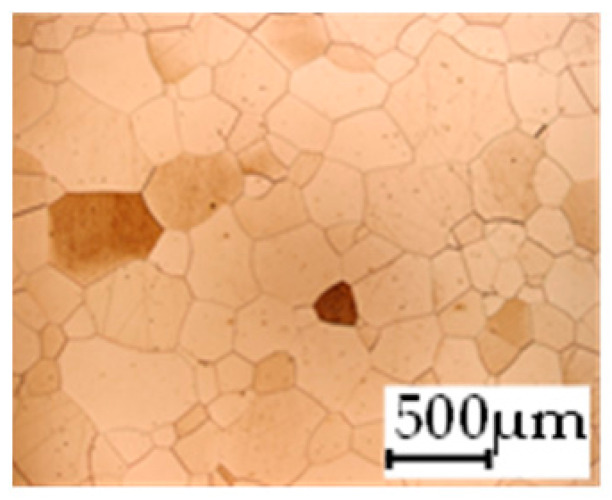
Surface	Middle	Surface	Middle	Surface	Middle
1g		1h		1i	
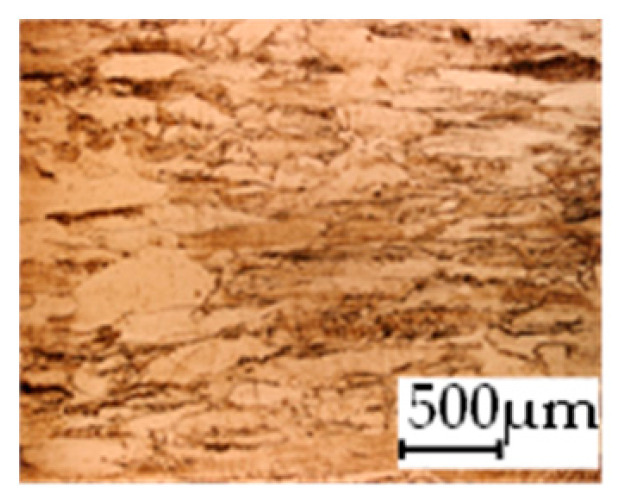	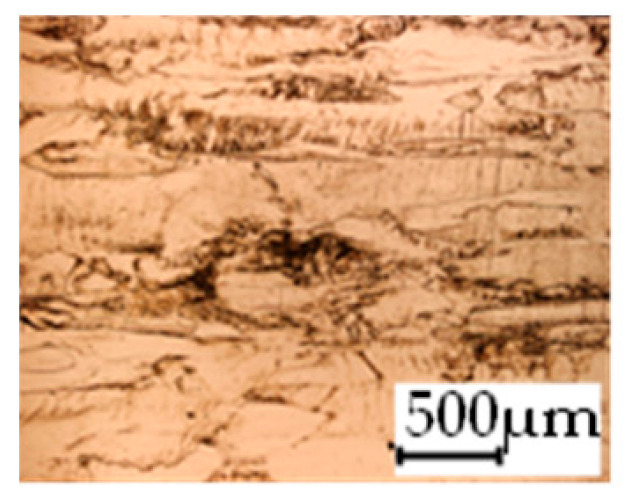	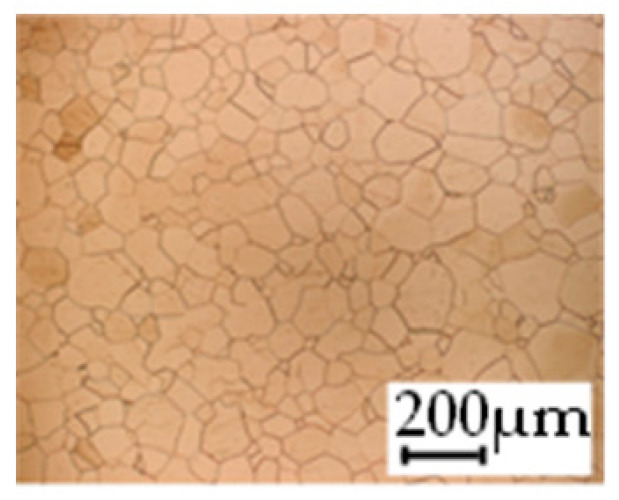	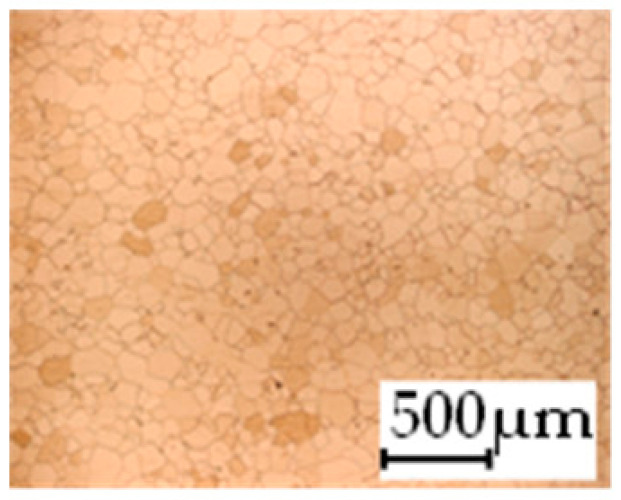	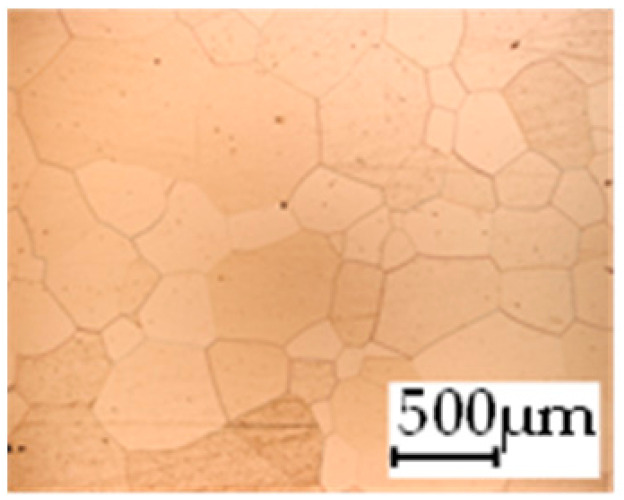	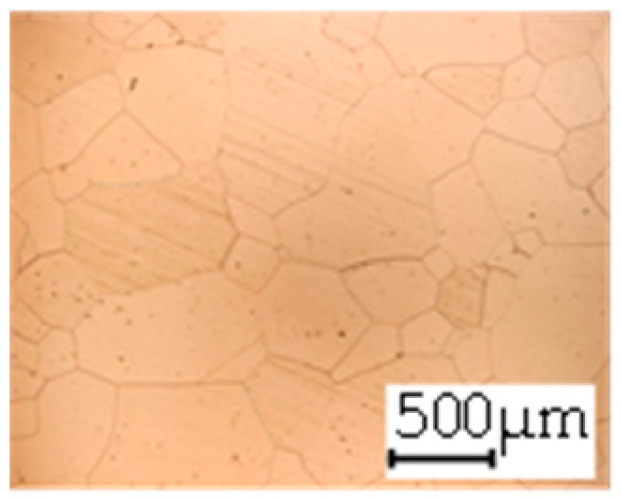
Surface	Middle	Surface	Middle	Surface	Middle
2a		2b		2c	
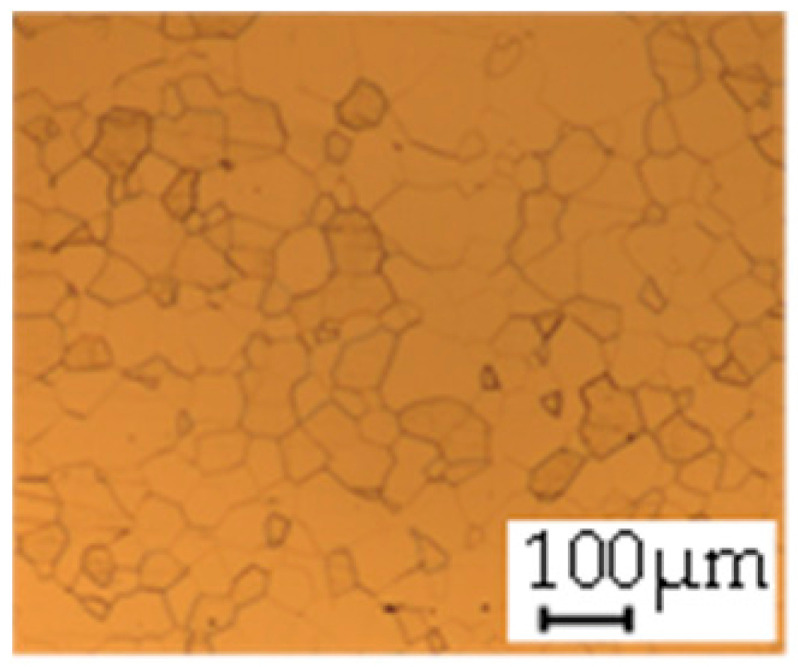	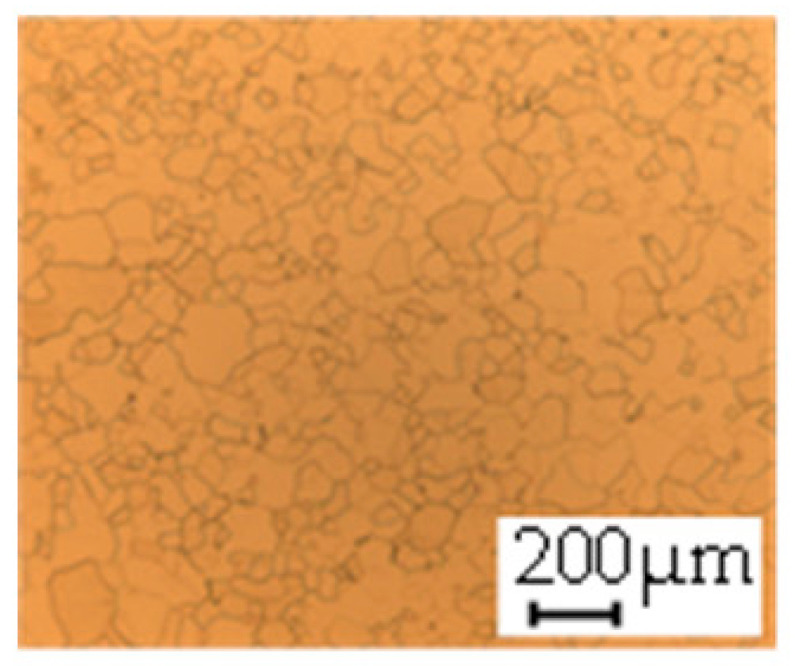	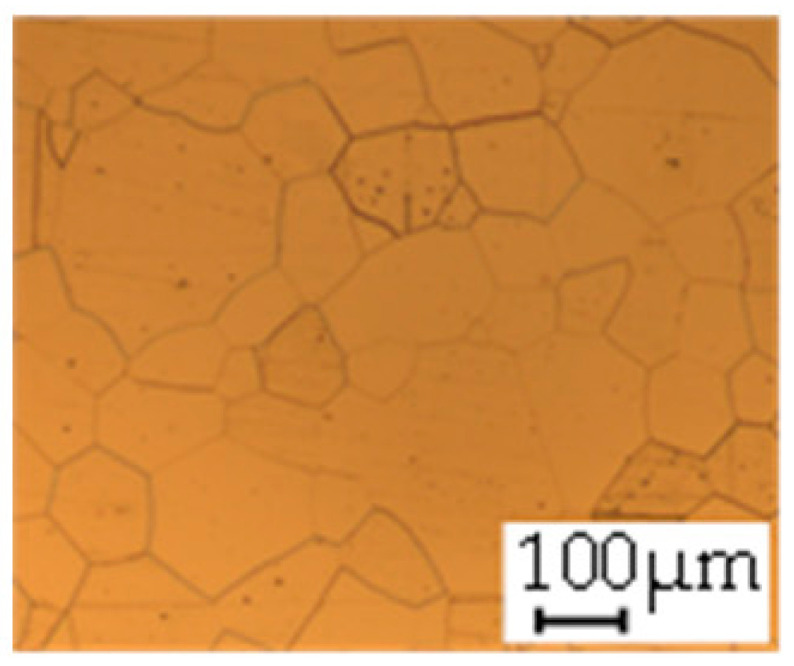	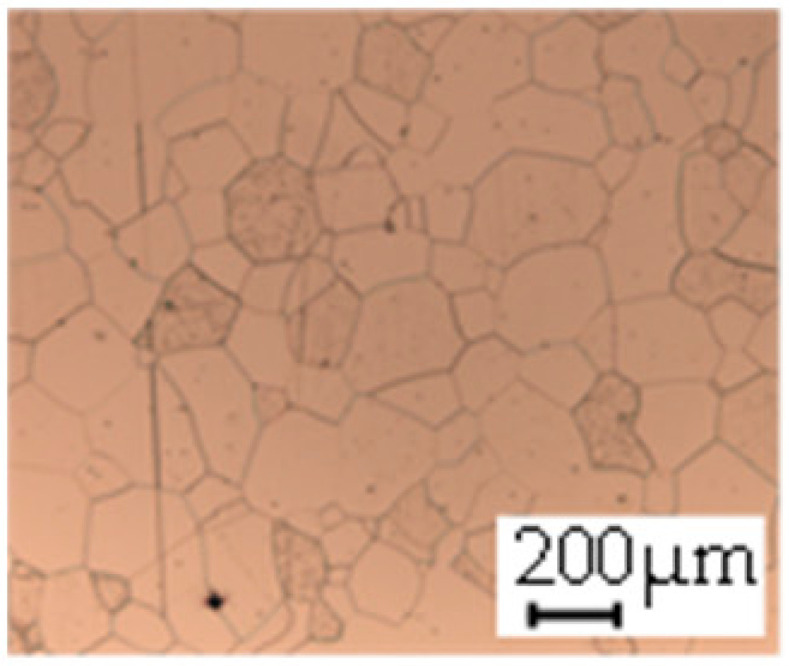	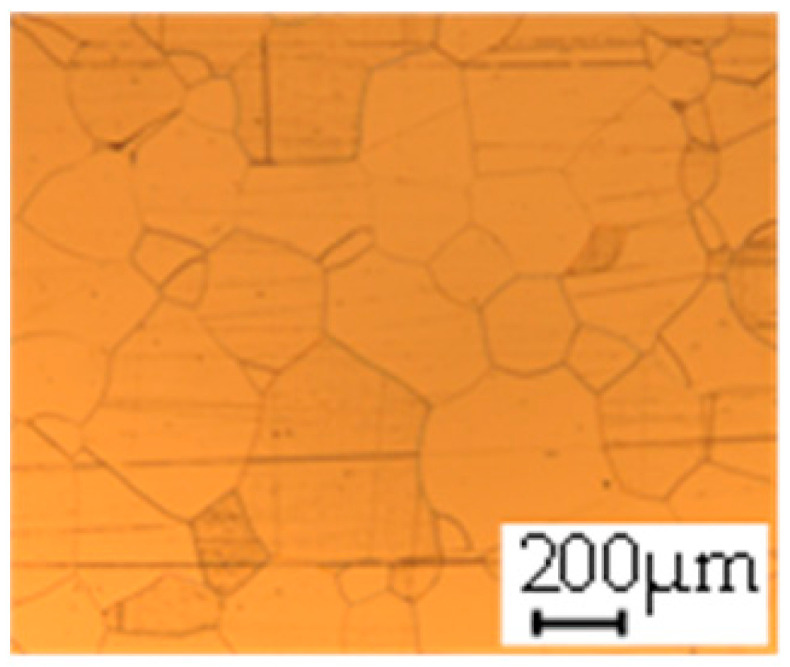	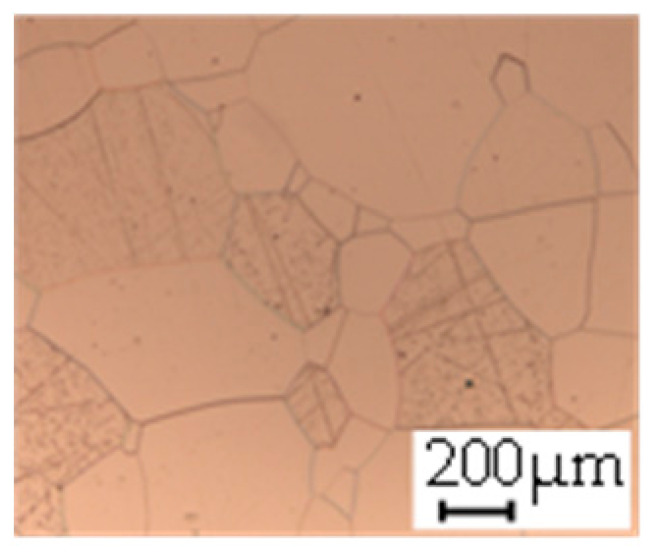
Surface	Middle	Surface	Middle	Surface	Middle
2d		2e		2f	
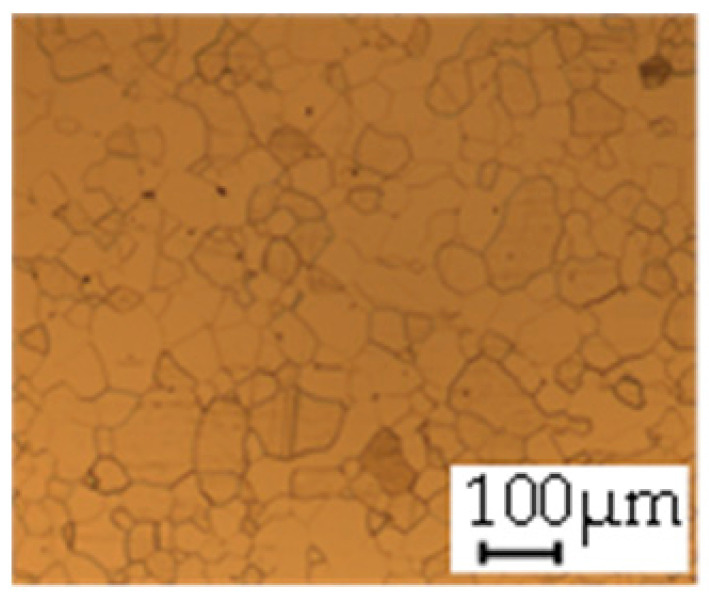	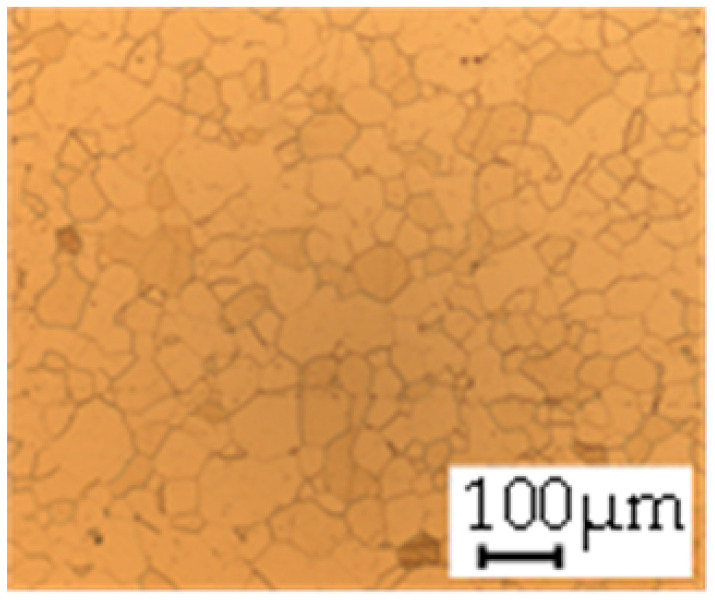	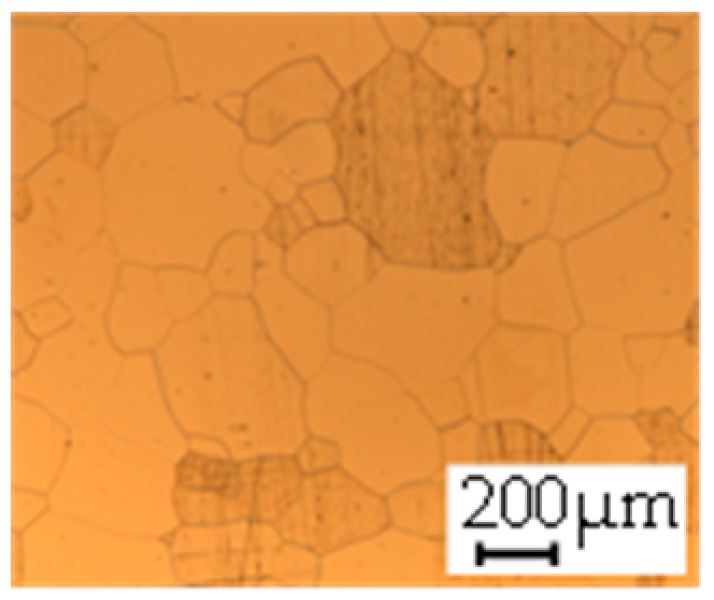	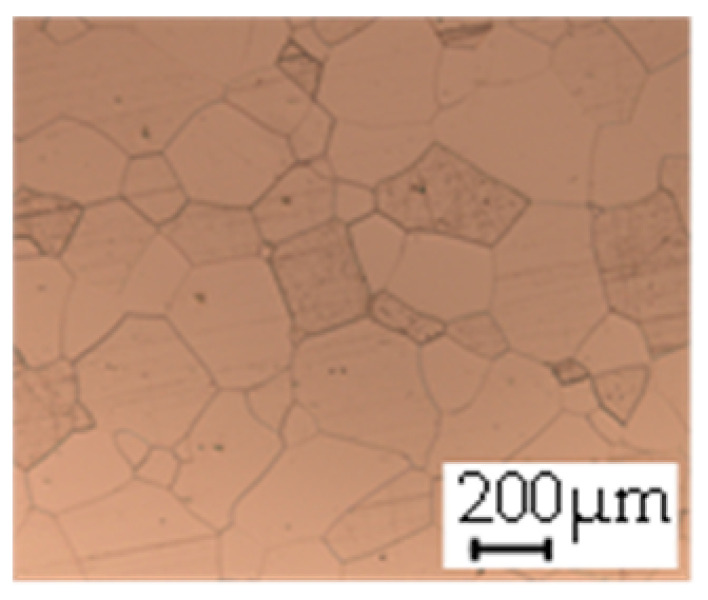	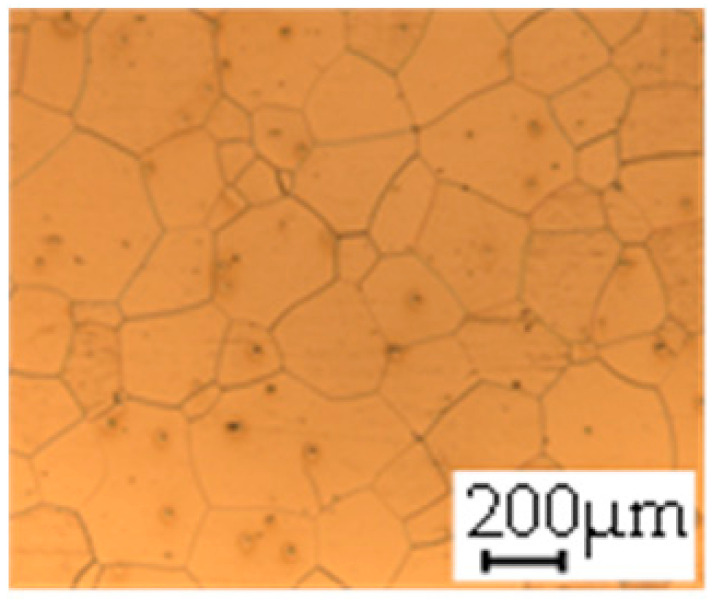	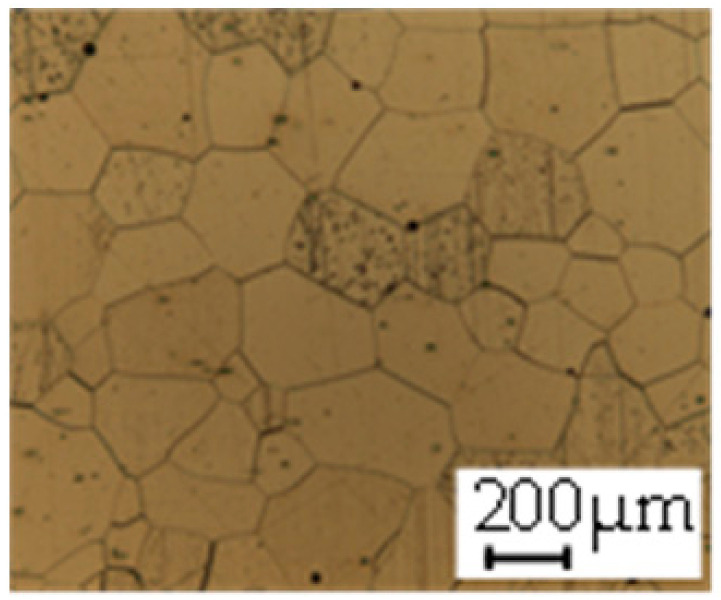
Surface	Middle	Surface	Middle	Surface	Middle
2g		2h		2i	
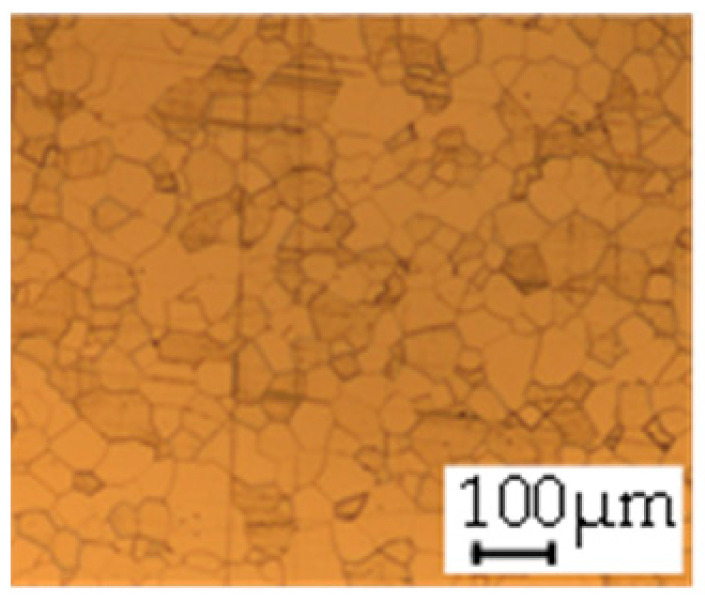	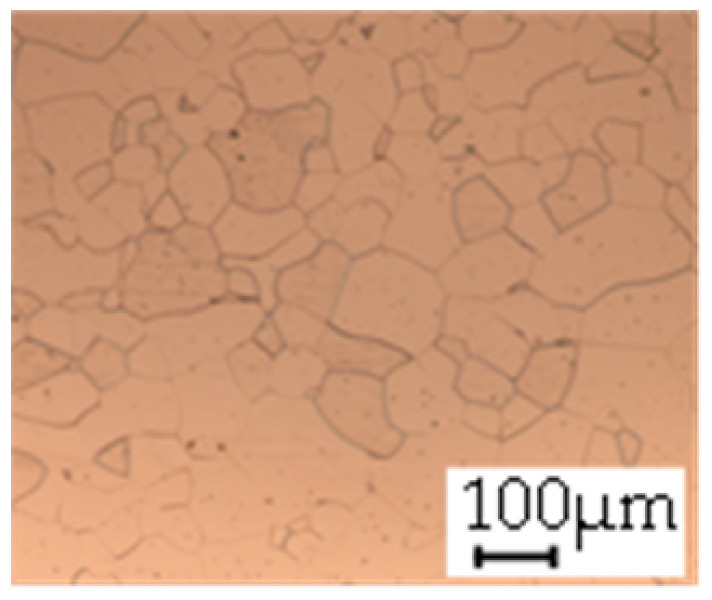	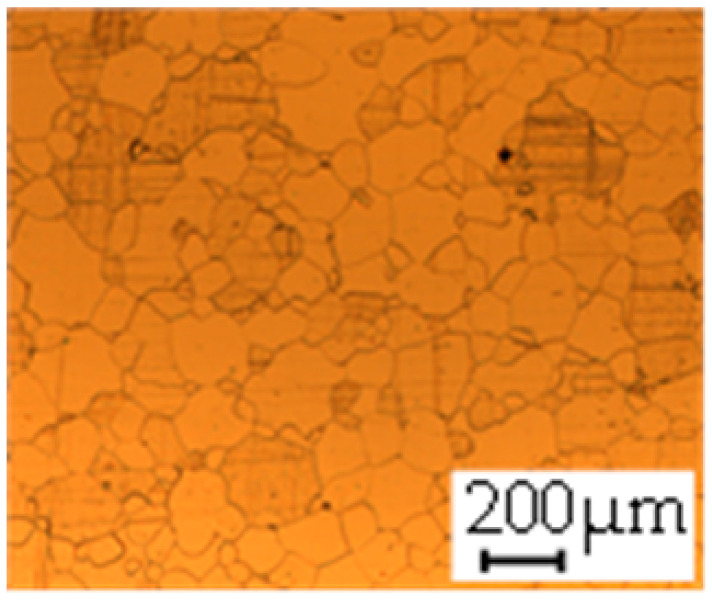	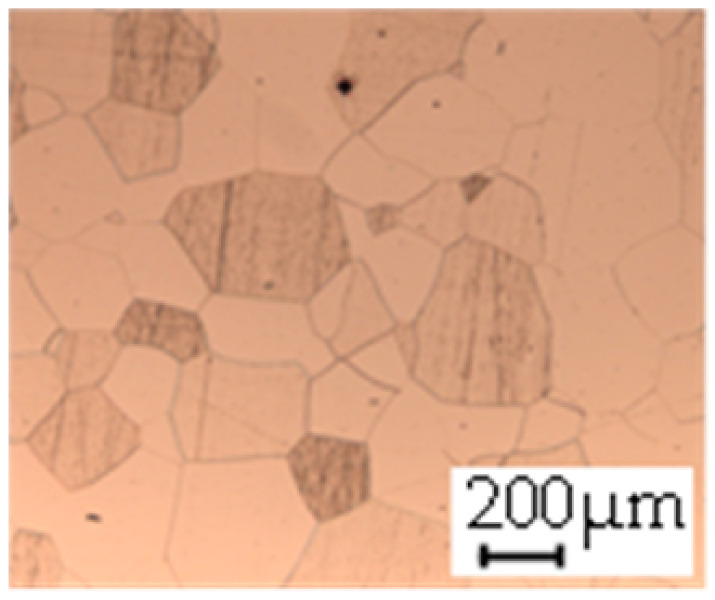	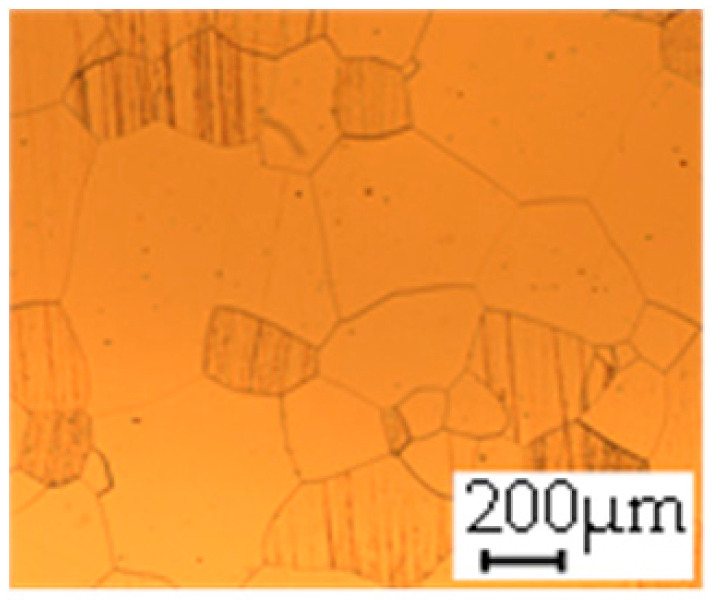	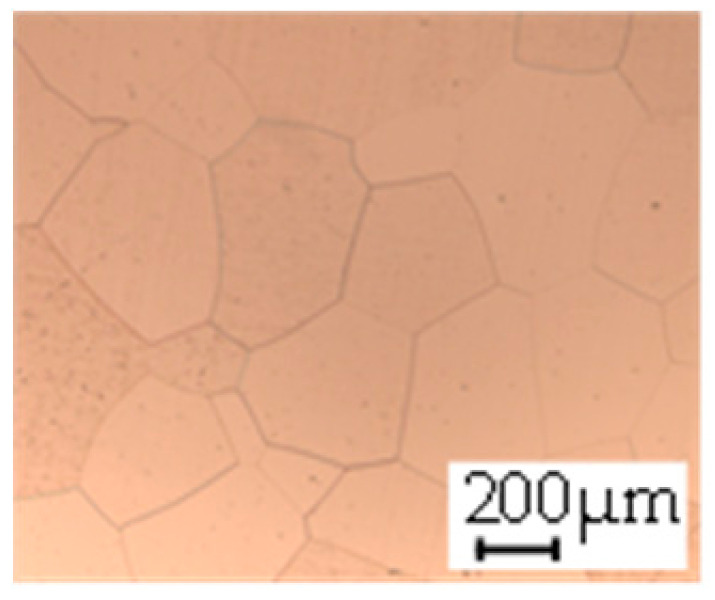
Surface	Middle	Surface	Middle	Surface	Middle
2j		2k		2l	
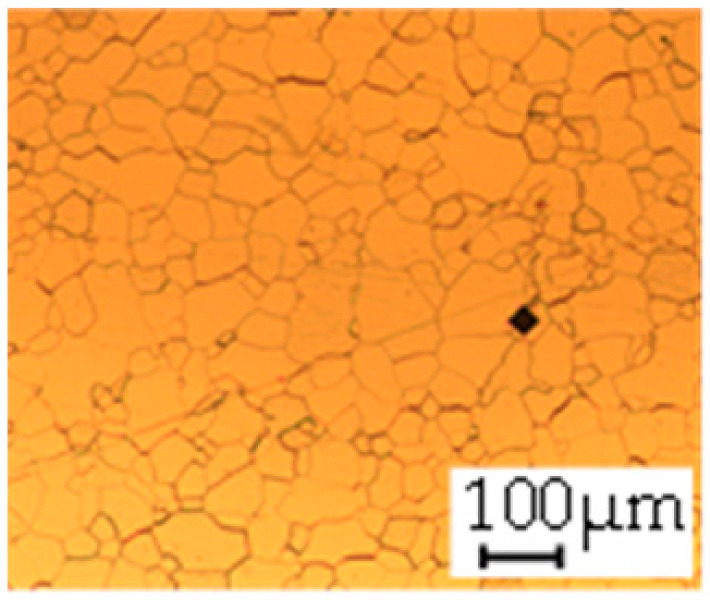	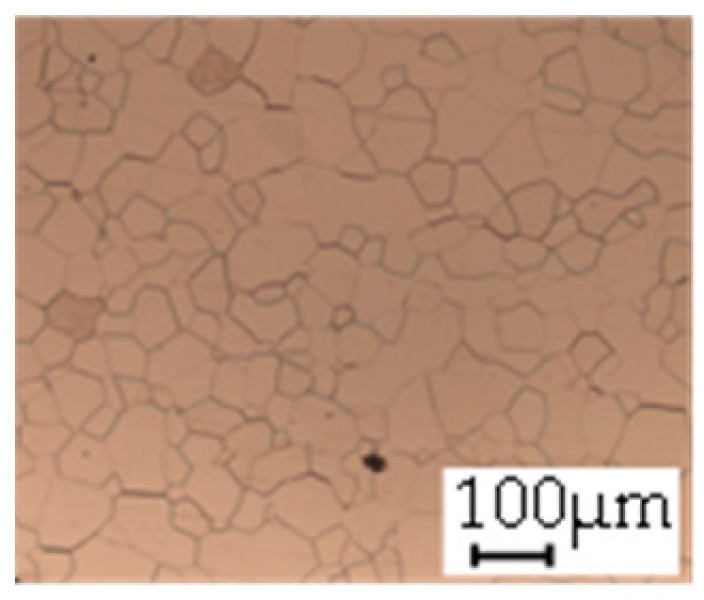	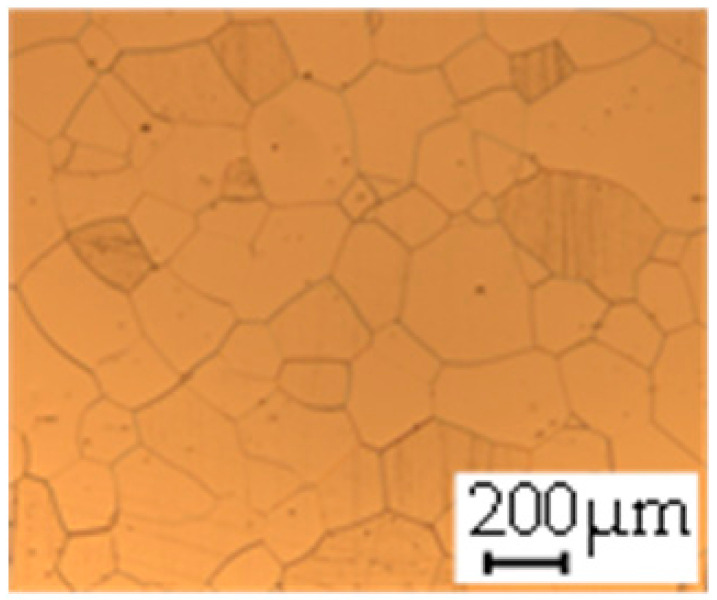	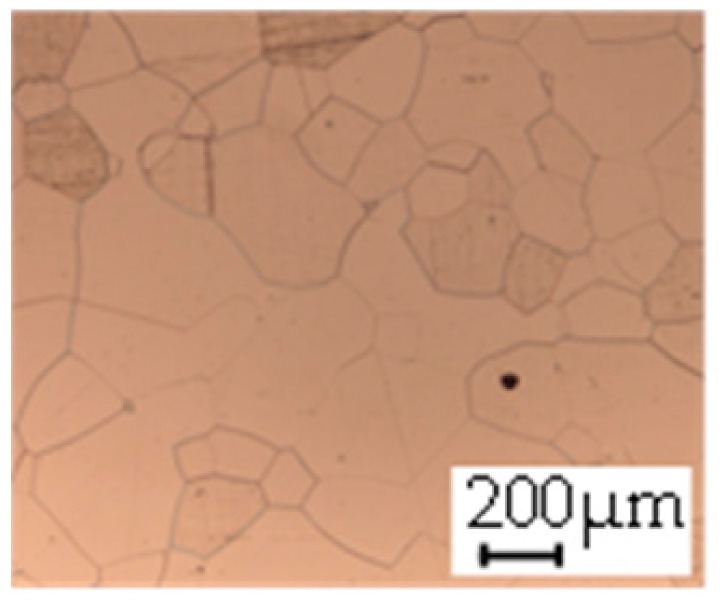	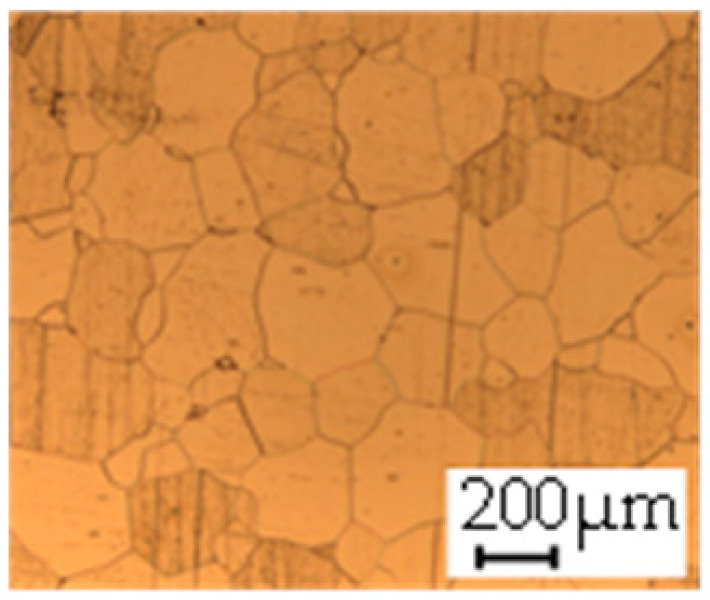	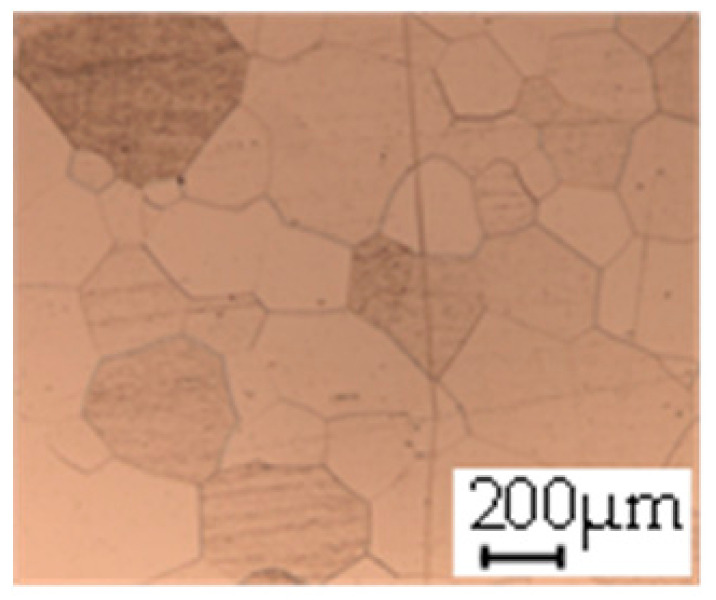
Surface	Middle	Surface	Middle	Surface	Middle

**Table 4:** Material parameters for the studied experimental steel grades.

	Thickness in mm	Alloying in wt %		Grain Size in µm			A-Parameter in °		
	Nominal	Si	Al	RD	TD	Mean	RD	TD	Mean
1a	0.50	2.38	0.44	41	44	43	31.27	34.88	32.96
1b	0.50	2.38	0.44	265	267	266	29.70	32.78	31.50
1c	0.25	2.38	0.44	15	16	15	36.59	39.23	33.24
1d	0.25	2.38	0.44	37	37	37	29.93	33.06	32.62
1e	0.25	2.38	0.44	65	65	65	29.92	33.44	32.82
1f	0.25	2.38	0.44	167	168	168	28.32	32.29	32.11
1g	0.50	2.38	0.44	15	15	15	36.30	38.50	32.85
1h	0.50	2.38	0.44	59	60	59	29.72	33.15	32.65
1i	0.50	2.38	0.44	367	298	332	27.95	30.73	30.42
2a	0.50	3.16	0.89	45	44	45	29.68	33.57	32.27
2b	0.50	3.16	0.89	108	107	107	30.04	32.07	31.62
2c	0.50	3.16	0.89	202	197	200	28.21	30.93	30.97
2d	0.25	3.16	0.89	31	31	31	29.78	33.69	32.24
2e	0.25	3.16	0.89	147	139	143	29.81	32.88	31.12
2f	0.25	3.16	0.89	175	165	171	29.69	30.83	30.20
2g	0.50	3.16	0.89	43	43	43	30.65	35.42	32.71
2h	0.50	3.16	0.89	138	135	137	31.08	35.18	32.45
2i	0.50	3.16	0.89	275	256	266	29.46	31.22	30.89
2j	0.25	3.16	0.89	39	37	38	27.95	33.88	31.08
2k	0.25	3.16	0.89	162	157	160	30.11	33.56	31.09
2l	0.25	3.16	0.89	177	173	175	29.44	30.28	29.35

**Table 5:** Loss parameters and material parameters for the tailor-made material design approach.

*a*_1_	α	β	$a_{2}\cdot 10^{-3}$	*a*_3_	*a*_4_	$a_{5}\cdot 10^{-3}$	*d*_sheet_ in mm	*d*_GS_ in µm	Si in wt %
0.28	1.3	0.45	0.345	0.3	1	0.6	0.3	80	3.2

## Data Availability

The datasets generated and analyzed during the current study may be available from the corresponding author on reasonable request.
